# Parent-of-origin effects on quantitative phenotypes in a large Hutterite pedigree

**DOI:** 10.1038/s42003-018-0267-4

**Published:** 2019-01-18

**Authors:** Sahar V. Mozaffari, Jeanne M. DeCara, Sanjiv J. Shah, Carlo Sidore, Edoardo Fiorillo, Francesco Cucca, Roberto M. Lang, Dan L. Nicolae, Carole Ober

**Affiliations:** 1 Department of Human Genetics, University of Chicago, Chicago, IL 60637 USA; 2 Committee on Genetics, Genomics, and Systems Biology, University of Chicago, Chicago, IL 60637 USA; 3 Department of Medicine, University of Chicago, Chicago, IL 60637 USA; 4 Department of Medicine, Northwestern University Feinberg School of Medicine, Chicago, IL 60611 USA; 5 Istituto di Ricerca Genetica e Biomedica (IRGB), CNR, Monserrato, 09042 Italy; 6 Dipartimento di Scienze Biomediche, Universita di Sassari, Sassari, 07100 Italy; 7 Department of Statistics, University of Chicago, Chicago, IL 60637 USA

## Abstract

The impact of the parental origin of associated alleles in GWAS has been largely ignored. Yet sequence variants could affect traits differently depending on whether they are inherited from the mother or the father, as in imprinted regions, where identical inherited DNA sequences can have different effects based on the parental origin. To explore parent-of-origin effects (POEs), we studied 21 quantitative phenotypes in a large Hutterite pedigree to identify variants with single parent (maternal-only or paternal-only) effects, and then variants with opposite parental effects. Here we show that POEs, which can be opposite in direction, are relatively common in humans, have potentially important clinical effects, and will be missed in traditional GWAS. We identified POEs with 11 phenotypes, most of which are risk factors for cardiovascular disease. Many of the loci identified are characteristic of imprinted regions and are associated with the expression of nearby genes.

## Abstract (web summary)

Sahar Mozaffari et al. conduct a parent of origin genome-wide association study in a large Hutterite pedigree to determine parent-of-origin effects on 21 phenotypes. They identify effects on 11 phenotypes (including single parent and opposite parental effects), most of which are associated with cardiovascular disease.

## Introduction

Genome-wide association studies (GWAS) typically treat alleles inherited from the mother and the father as equivalent, although variants can affect traits differently depending on whether they are maternal or paternal in origin. In particular, parent-of-origin effects (POEs) can result from imprinting, where epigenetic modifications allows for differential gene expression on homologous chromosomes that is determined by the parental origin of the chromosome. Mutations in imprinted genes or regions can result in diseases. For example, two very different diseases, Prader-Willi Syndrome and Angelman Syndrome are due to loss of function alleles in genes within an imprinted region on chromosome 15q11-13. Inheriting a loss of function mutation for the *SNRPN* gene from the father results in Prader-Willi Syndrome but inheriting a loss of function mutation for the *UBE3A* gene from the mother results in Angelman Syndrome^1,2^. Long noncoding RNA genes at this and other imprinted regions act to silence (i.e., imprint) genes in *cis*. Imprinted genes are often part of imprinted gene networks, suggesting regulatory links between these genes^3–5^. More than 150 imprinted genes have been described in humans^6^, but there are likely many other, as yet undiscovered, imprinted loci.

Previous studies have utilized pedigrees to test maternal and paternal alleles separately for association with phenotypes or with gene expression to uncover new imprinted loci^6–10^. Kong et al.^7^ discovered one locus associated with breast cancer risk only when the allele is inherited from the father and another locus associated with type 2 diabetes risk only when the allele is inherited from the mother. Garg et al.^8^ reported parent-of-origin *cis*-eQTLs with known or putative imprinted genes affecting gene expression. Two additional studies by Zoledziewska et al.^11^ and Benonisdottir et al.^6^ identified opposite POEs on adult height at known imprinted loci. Both studies reported associations with variants at the *KCNQ1* gene, and one showed additional opposite POEs with height at two known imprinted loci (*IGF2-H19* and *DLK1-MEG3*)^6^. These studies provide proof-of-principle that alleles at imprinted loci can show POEs, some with opposite effects, with common phenotypes.

Many existing studies and methods identify parent-of-origin effects use case/parent trios or case/mother duos^12–16^. Similar to Kong et al.^7^, our method does not require data on the parent and only uses the parent-of-origin informative alleles, which were assigned and phased using PRIMAL^17^. In contrast to Kong et al.^7^ which used binary traits, our method tests for parent-of-origin effects on quantitative traits, similar to Benonisdottir et al.^6^, which tested for parent-of-origin effects on height.

No previous study has included a broad range of human quantitative phenotypes or has studied genome-wide variants with effects in different directions depending on the parent-of-origin. To address this possibility, we develop a statistical model that directly compares the effects of the maternal and paternal alleles to identify effects that are different, including those that are opposite. We apply this model in a study of 21 common quantitative traits that were measured in the Hutterites, a founder population of European descent for which we have phased genotype data^17^. We identify variants with maternally inherited or paternally inherited effects only and variants with opposite POEs. Some of the identified regions have characteristics similar to known imprinted genes. Overall, we show that this model can identify putative imprinted regions with POEs for a broad range of clinically relevant quantitative phenotypes.

## Results

### Genome-wide association studies (GWAS)

We first performed standard GWAS of 21 traits in the Hutterites (Supplementary Table 1). These studies identified one genome-wide significant association (*p* < 5 × 10^–8^) with each of five of the 21 traits: low-density lipoprotein level (LDL)-cholesterol, triglycerides, carotid artery intima media thickness (CIMT), left ventricular mass index (LVMI), and monocyte count. The results of all 21 GWAS are summarized in Supplementary Table 2 and Supplementary Fig. 1. Results for all variants for all GWAS are deposited in dbGaP (phs000185).

### Parent-of-origin GWAS

We considered two possible mechanisms of POEs. In the first, the effect size of one parent’s allele is close to zero and the effect size of the other parent’s allele is different from zero. For these cases, we performed a paternal only or maternal only GWAS. In other cases, the maternal and paternal alleles may both have effect sizes different from zero, but the effects are significantly different from each other or opposite in direction. To detect these types of POEs, we developed a model that tests for differences between parental effects (see Methods). This model is especially powerful to identify variants with parental effects in opposite directions.

*Maternal and paternal GWAS*: Using the same phenotypes, genotypes, pedigree, and criteria for significance as in the standard GWAS, we tested for maternal and paternal effects on each trait by testing each parentally inherited allele with the trait of interest, similar to previous studies^7,8,11^. Variants were considered to have POEs if they had a *p*-value less than 5 × 10^–8^ in only one parent and were not significant in the standard GWAS (i.e., the LDL association on chromosome 19 and the triglycerides association chromosome 11 were not considered to have POEs; see Supplementary Table 1). The most significant parent-of-origin associations are summarized in Table 1. All significant results of the parent-of-origin maternal and paternal GWAS for all 21 phenotypes are included in Supplementary Data 1 and 2.

**Table 1:** Phenotypes with significant single parent-of-origin associations

Phenotype	rsid (effect allele/other allele)	chr:loc	Variant location	Nearest gene	MAF	*N*	Beta (SE)	Paternal GWAS *p*-value	Maternal GWAS *p*-value	Standard GWAS *p*-value
**A** Maternal associations										
Age of menarche	rs7184983 (A/G)	16:56554709	Upstream	*BBS2*	0.059	336	0.862 (0.154)	5.01E–01	3.11E–08	6.75E–03
CIMT	rs4077567 (G/A)	2:216703202	Intronic	*LINC00607* (noncoding RNA gene)	0.30	429	0.047 (0.008)	5.72E–01	3.02E–08	4.21E–06
FEV_1_	rs9849387 (A/G)	3:77764243	Intergenic	*ROBO2*	0.39	1029	−0.089 (0.015)	3.87E–01	4.10E–09	4.38E–04
	rs6791779 (C/G)	3:74996505	Intergenic	*MIR4444-1* (noncoding RNA gene)	0.24	879	−0.102 (0.021)	6.88E–02	1.48E–08	4.52E–02
LVMI	rs574232282 (G/A)	1:41662388	Intronic	*SCMH1*	0.018	537	0.239 (0.042)	5.52E–01	1.39E–08	1.05E–03
**B** Paternal Associations										
LDL	rs12024326 (A/G)	1:227146433	Intronic	*ADCK3*	0.175	686	−0.295 (0.048)	8.06E–10	4.21E–01	4.24E–05
	rs4843650 (A/G)	16:87683486	Intronic	*JPH3*	0.448	621	0.211 (0.036)	6.57E–09	2.21E–01	1.50E–04
SBP	rs1536182 (A/G)	13:46275415	Upstream	*LINC01055* (noncoding RNA gene)	0.2	684	−0.028 (0.005)	1.53E–08	1.78E–01	6.93E–04
Total cholesterol	rs113588203 (G/T)	1:228979156	Intergenic	*RHOU*	0.099	703	−0.341 (0.060)	1.76E–08	7.43E–02	8.08E–03

The most significant variant (*p* < 5 × 10^–8^) at each locus for the (A) maternal and (B) paternal associations associated with each phenotype is shown

Overall, seven phenotypes had genome-wide significant parent-of-origin associations: four in the maternal only GWAS and three in the paternal only GWAS. Three cardiovascular disease (CVD)-associated phenotypes (age at menarche, CIMT, LVMI) and one lung function phenotype (forced expiratory volume in 1 second [FEV_1_]) were associated with maternally inherited alleles only.

**Fig. 1:**
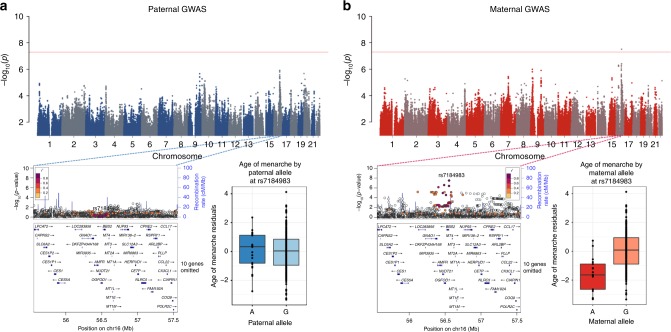
Maternal and paternal GWAS results for age of menarche. The top panel shows the Manhattan plots from the paternal (**a**) and maternal (**b**) GWAS. LocusZoom plots for both GWAS are shown in the lower panel for the associated region in the GWAS. Box plots show the distribution of age of menarche residuals (*y*-axes) by the corresponding maternal and paternal alleles at this SNP (*x*-axes). The horizontal bar of the boxplot shows the median, the box delineates the first and third quartile, and the whiskers show + /−1.5 × IQR

A maternally inherited allele at rs7184983 (G) on chromosome 16 was associated with younger age of menarche (*p* = 3.11 × 10^–8^) (Fig. 1). This SNP, rs7184983, is located upstream of the *BBS2* gene and is associated with increased expression of *OGFOD1* in transformed fibroblast cells and tibial nerve (*p* = 6.3 × 10^–10^)^18^. The maternally inherited allele at rs4077567 (G) on chromosome 2 was associated with decreased CIMT (*p* = 3.02 × 10^–8^) (Supplementary Fig. 2). This SNP is in the intron of a long intergenic noncoding gene, *LINC00607*, that is expressed in aorta, coronary, and tibial artery, all tissues potentially relevant to CIMT and atherosclerosis^18^. A maternally inherited allele at rs574232282 (G) in the intron of *SCMH1* on chromosome 1 was associated with increased LVMI (*p* = 1.39 × 10^–8^) (Supplementary Fig. 3). *SCMH1* is expressed in aorta, coronary, and tibial artery^18^. SCMH1 protein associates with the polycomb group multiprotein complexes required to maintain the transcriptionally repressive state of certain genes^18^. Lastly, maternally inherited alleles at rs9849387 (A) and rs6791779 (C) on chromosome 3 were both associated with reduced FEV_1_ (*p* = 4.10 × 10^–9^ and 1.48 × 10^–8^, respectively) (Supplementary Fig. 4). The nearest gene to rs9849387 is *ROBO2* (65 kb, downstream), which is expressed in the lung as well as in brain, and ovary^18^. The nearest gene to rs6791779 is MIR4444-1(267 kb) whose expression has not been characterized.

**Fig. 2:**
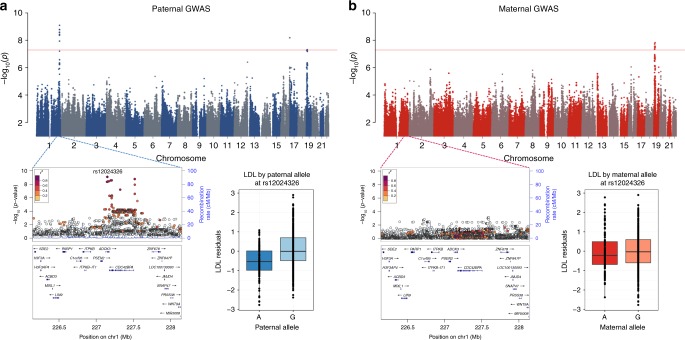
Maternal and paternal GWAS results for LDL cholesterol. The top panel shows the Manhattan plots from the paternal (**a**) and maternal (**b**) GWAS. LocusZoom plots for both GWAS are shown in the lower panel for the associated region in the GWAS. Box plots show the distribution of LDL residuals (*y*-axes) by the corresponding maternal and paternal alleles at this SNP (*x*-axes). The horizontal bar of the boxplot shows the median, the box delineates the first and third quartile, and the whiskers show + /−1.5 × IQR

Three other CVD-related phenotypes (systolic blood pressure, LDL-C, and total cholesterol) had associations with paternally inherited alleles only. The paternally inherited allele at rs12024326 (A) on chromosome 1 was associated with lower LDL-cholesterol levels (*p* = 8.06 × 10^–10^) (Fig. 2). rs12024326 is in the intron of gene *ADCK3*, and the same allele was associated with increased expression of *ADCK3* in whole blood (*p* = 2.5 × 10^–11^), as well as decreased expression of a neighboring gene, *CDC42BPA* in brain (cerebellum), heart (left ventricle), esophagus, and tibial artery (*p* = 3 × 10^–11^)^18^. The paternal G allele at rs4843650 on chromosome 16 was associated with increased LDL-C and is located in the intron of *JPH3*, which is expressed predominantly in the brain^18^. A SNP on chromosome 13 (rs1536182) was associated with systolic blood pressure levels when it was inherited from the father (Supplementary Fig. 5). The paternally inherited A allele at this SNP was associated with decreased systolic blood pressure, as well as decreased expression of its closest gene, *LINC01055*, a long intergenic noncoding gene, in testis (*p* = 2.5 × 10^–07^)^18^. A paternally inherited allele at rs113588203 (G) on chromosome 1 was associated with lower total cholesterol (*p* = 1.76 × 10^–8^) (Supplementary Fig. 6). This SNP is intergenic between *RHOU* (96 kb, downstream), which is expressed across multiple tissues, and *MIRR4454* (331 kb), which is expressed in adipose, kidney and heart tissues^18^.

*GWAS for differential parent-of-origin effects*: Because some imprinted regions include genes that have both maternal and paternal specific tissue expression, we next tested for such differential effects with these 21 phenotypes. In these analyses, we compared the effect and direction of the association between maternal and paternal alleles to identify variants that have different effects, including opposite effects, on the phenotype. Such loci would be completely hidden in standard GWAS in which paternally and maternally inherited alleles are combined. These opposite effect GWAS revealed 11 independent loci with opposite POEs for nine different traits, at least six of which are associated with CVD risk (Table 2 and Supplementary Fig. 7). All significant results of the parent-of-origin GWAS for all 21 phenotypes are included in Supplementary Data 3.

**Table 2:** Significant opposite parent-of-origin effect GWAS associations

Phenotype	rsid	chr:loc	Variant location	Nearest gene	MAF	*β*_M _– *β*_P_ (SE)	Opposite effect GWAS	Paternal GWAS		Maternal GWAS		Standard GWAS *p*-value
								*p*-value	Beta (SE)	*p*-value	Beta (SE)	
Age of menarche	rs57878386	16:62201228	Intergenic	*CDH8*	0.17	−0.654 (0.109)	5.27E–09	5.20E–06	0.391 (0.085)	1.85E–05	−0.368 (0.085)	8.68E–01
BMI	rs77785972	5:97415767	Intergenic	*LINC01340* (noncoding RNA gene)	0.025	0.154 (0.025)	5.12E–10	5.84E–07	−0.094 (0.019)	1.58E–05	0.081 (0.019)	5.39E–01
	rs17605739	6:22962798	Intronic	*RP1-209A6.1* (noncoding RNA gene)	0.17	0.053 (0.010)	3.01E–08	6.99E–05	−0.032 (0.008)	1.42E–06	0.034 (0.007)	1.56E–01
Eosinophil count	rs2355879	1:18732860	Intergenic	*IGSF21*	0.14	0.091 (0.016)	1.69E–08	5.83E–08	−0.065 (0.012)	5.59E–04	0.043 (0.012)	2.53E–01
FEV_1_	rs12714812	3:74813002	Intergenic	*CNTN3*	0.45	−0.119 (0.021)	4.52E–08	1.78E–03	0.052 (0.017)	6.35E–06	−0.073 (0.016)	9.58E–01
LDL	rs1032596	16:86281537	Intronic	*LINC01081* (noncoding RNA gene)	0.30	−0.310 (0.056)	3.69E–08	1.05E–06	0.201 (0.041)	4.56E–04	−0.148 (0.042)	2.71E–01
LVMI	rs16853098	2:168013281	Intronic	*XIRP2*	0.12	−0.091 (0.053)	4.18E–08	5.29E–06	0.064 (0.014)	2.04E–04	−0.048 (0.013)	9.26E–01
Neutrophil count	rs142030841	18:34371947	Intronic	*TPGS2*	0.042	−0.224 (0.041)	4.40E–08	2.25E–03	0.078 (0.025)	1.30E–07	−0.188 (0.035)	5.77E–01
Triglycerides	rs7525463	1:218860879	Intronic	*MIR548F3* (noncoding RNA gene)	0.16	−0.401 (0.071)	2.51E–08	1.14E–03	0.195 (0.060)	5.52E–08	−0.267 (0.049)	2.84E–02
Total cholesterol	rs7033776	9:36704465	Intergenic	*MELK*	0.41	0.230 (0.041)	4.12E–08	5.60E–08	−0.183 (0.034)	2.28E–03	0.099 (0.032)	6.70E–02

The most significant variant at each locus for each phenotype is shown. *β*_M_ – *β*_P_ represents difference in parental effect size

**Fig. 3:**
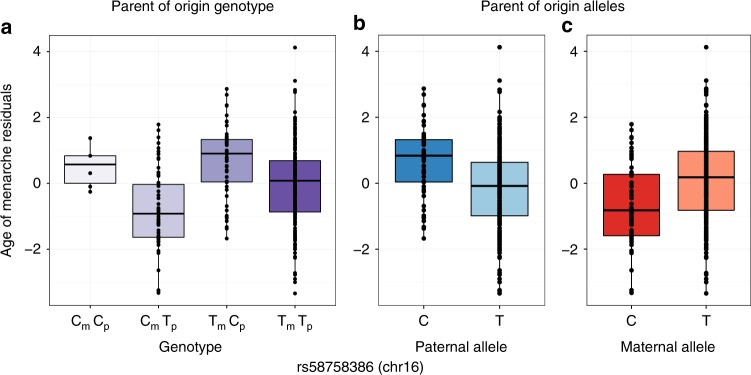
Opposite effect parent-of-origin GWAS result for age of menarche. Box plots of age of menarche residuals (*y*-axes) are shown for each of the four genotypes (on the *x*-axis) (**a**), and for paternal (**b**) and maternal (**c**) alleles. The maternal C allele is associated with decreased and maternal T allele with increased age of menarche. The paternal C allele is associated with increased and the paternal T allele with decreased age of menarche. The horizontal bar of the boxplot shows the median, the box delineates the first and third quartile, and the whiskers show + /−1.5 × IQR

**Fig. 4:**
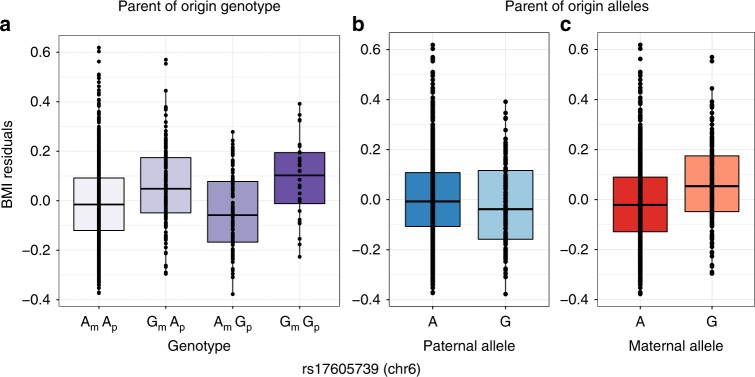
Opposite effect parent-of-origin GWAS result for BMI. Box plots of BMI residuals (*y*-axes) for each of the four genotypes (**a**), and for paternal (**b**) and maternal (**c**) alleles. The paternal A allele is associated with increased and the paternal G allele with decreased BMI. The maternal A allele is associated with decreased and maternal G allele with increased BMI. The paternal A allele is associated with increased and the paternal G allele with decreased BMI. The horizontal bar of the boxplot shows the median, the box delineates the first and third quartile, and the whiskers show + /−1.5 × IQR

A locus on chromosome 16, near the *CDH8* gene (128 kb, upstream), was associated with opposite POEs with age of menarche (Fig. 3). *CDH8* is highly expressed in the brain, as well as in the aorta artery and pituitary gland. Two loci on chromosomes 5 and 6 were associated with opposite POEs on body mass index (BMI) (Fig. 4). The most significant variant on chromosome 5 (rs77785972) is near a long intergenic noncoding gene, *LINC01340* (409 kb, downstream), whose expression has not been well characterized. The SNP on chromosome 6 (rs17605739) is also in a long intergenic noncoding gene, *RP1-209A6.1*, which is expressed in low levels in the tibial artery, bladder, spleen, lung, pituitary gland, as well as testis.

A SNP on chromosome 16 (rs1032596) was associated with opposite POEs on LDL-cholesterol (Supplementary Fig. 8). This SNP lies in the intron of another long noncoding RNA gene, *LINC01081*, which has been suggested to be imprinted because its downstream genes have also been shown to have parent- and tissue-specific activity^19^. A region on chromosome 2 has opposite effects associated with LVMI (Supplementary Fig. 9). The associated SNPs are in the intron of *XIRP2*, a cardiomyopathy associated protein that is expressed in skeletal muscle and heart left ventricle, suggesting that this gene could play a role in determining left ventricular mass^20–22^. In addition, the most significant SNP at this region, rs17616252 (and multiple SNPs in LD) is a strong eQTL (*p* = 1.8 × 10^–13^) for the gene *XIRP2* in skeletal muscle, *XIRP2-AS1* in testis, and *B3GALT1* in transformed fibroblast cells (*p* = 9.9 × 10^–09^)^18^. Four variants in a region on chromosome 1 in a microRNA gene, *MIR548F3*, were associated with opposite POEs on triglyceride levels (Supplementary Fig. 10). The expression of *MIR548F3* has not been characterized. SNP rs7033776 near *MELK* (27 kb, downstream) on chromosome 9 was associated with opposite effects on total cholesterol (Supplementary Fig. 11). *MELK* is expressed in the colon and esophagus in addition to transformed lymphocytes and fibroblasts^18^.

Nine linked variants on chromosome 1 were associated with opposite POEs of blood eosinophil count (Supplementary Fig. 12). These variants are near the gene *IGSF21* (27 kb, downstream) which is a member of the immunoglobulin superfamily and likely acts as a receptor in immune response pathways^23^. A variant on chromosome 3, rs12714812, was associated with opposite POEs for FEV_1_ (Supplementary Fig. 13). This variant has been shown to regulate the expression of a gene *CNTN3* (45 kb, upstream) in heart and brain (*p* = 2.2 × 10^–20^)^18^. Studies in mice have suggested that this gene is imprinted and maternally expressed in the murine placenta^24^. Variant rs142030841 in the intron of the gene *TPGS2* on chromosome 18 has opposite POEs with neutrophil levels (Supplementary Fig. 14). This SNP is an expression quantitative trait locus (eQTL) for the noncoding RNA gene *RP11-95O2.5* in skin, testis, breast, thyroid and adipose tissue (*p* = 4.5 × 10^–09^), for *CELF4* in tibial nerve and lung (*p* = 6 × 10^–11^), and for *TPGS2* in tibial artery and transformed fibroblast cells (*p* = 1.5 × 10^–05^)^18^.

### Parent-of-origin effects on gene expression

To determine if any of the associated variants also showed POEs on gene expression in the Hutterites, we used RNA-seq gene expression data from lymphoblastoid cell lines (LCLs) collected from 430 of the individuals in the GWAS sample. We first tested for association of maternal and paternal variants with genes detected as expressed in the LCLs and whose transcript start site was within 1 Mb of each associated SNP (Supplementary Table 3). There were no significant associations after multiple testing correction, similar to a previous study^6^. However, because we considered this to be exploratory analyses, we show results for the five most significant parent-of-origin eQTLs (Table 3). We next used the opposite effect model for each SNP in Table 2 and expression of all genes that were detected as expressed in LCLs and whose transcript start site was within 1 Mb of the associated SNP (Supplementary Table 4). This resulted in 57 tests (1 SNP for each of 8 phenotypes, and 57 genes). The five most significant opposite effect eQTLs, none of which passed the Bonferroni threshold of 8.77 × 10^–4^, are shown in Table 4. The most significant opposite effect eQTL was for *POLR1E* expression with the SNP on chromosome 9 (rs7033776) that was associated with total cholesterol (opposite effect eQTL *p* = 9.86 × 10^–4^) (Supplementary Fig. 15). *POLR1E* is involved in the purine metabolism pathway as well as DNA-directed polymerase activity. The same SNP, rs7033776, had modest opposite effects with the expression of three other genes in the region (*PAX5*, *FBXO10*, and *FRMPD1*), a signature consistent with an imprinted region. Another SNP with opposite POEs on LVMI, rs16853098, was an opposite effect eQTL for *STK39*, a gene that has been previously associated with hypertension^25^.

**Table 3:** Parent-of-origin eQTLs in LCLs

Phenotype	Sample size	rsid	chr:loc	Gene	Beta (SE)	Maternal eQTL *p*-value	Paternal eQTL *p*-value
**A** Maternal associations							
CIMT	334	rs4077567	1:216703202	*ABCA12*	0.039 (0.017)	2.14E–02	1.53E–02
Age of menarche	336	rs7184983	16:56554709	*POLR2C*	−0.085 (0.039)	2.91E–02	7.93E–01
Age of menarche	336	rs7184983	16:56554709	*SLC12A3*	−0.064 (0.031)	3.77E–02	2.28E–01
CIMT	334	rs4077567	1:216703202	*RPL37A*	0.030 (0.016)	5.72E–02	5.90E–01
LVM	457	rs74232282	1:41662388	*SMAP2*	1.40 (0.159)	5.82E–02	1.12E–01
**B** Paternal associations							
Total cholesterol	352	rs113588203	1:228979165	*HIST3H2A*	0.560 (0.308)	8.81E–01	6.85E–02
Total cholesterol	352	rs113588203	1:228979165	*SPHAR*	0.073 (0.047)	6.01E–01	1.20E–01
LDL	352	rs1110603	16:87687317	*MAP1LC3B*	−0.024 (0.015)	4.35E–01	1.25E–01
LDL	352	rs1110603	16:87687317	*FBXO31*	−0.027 (0.018)	1.56E–01	1.36E–01
Total cholesterol	357	rs113588203	1:228979165	*RAB4A*	−0.039 (0.028)	6.16E–01	1.59E–01

The most significant SNP for each phenotype (Table 1) was tested for association with gene expression for genes with TSS within 1 Mb of the SNP. The effect sizes correspond to the maternal (A) or paternal (B) effect sizes. Only the five most significant SNPs are listed for each parental eQTL

**Table 4:** Opposite parent-of-origin eQTLs in LCLs

Phenotype	Sample size	rsid	chr:loc	Gene	*β*_M _– *β*_P_ (SE)	Opposite effect *p-*value
Total cholesterol	381	rs7033776	9:36704465	*POLR1E*	0.0603 (0.399)	9.86E–04
Total cholesterol	381	rs7033776	9:36704465	*PAX5*	0.0608 (0.0253)	0.0162
Total cholesterol	381	rs7033776	9:36704465	*FBXO10*	0.0789 (0.0337)	0.019
LVMI	355	rs16853098	2:168013281	*STK39*	−0.238 (0.124)	0.055
Total cholesterol	381	rs7033776	9:36704465	*FRMPD1*	0.185 (0.0988)	0.060

The most significant SNP for each phenotype (Table 2) was tested for opposite effect eQTLs with genes with TSS within 1 Mb of the SNP. The effect size corresponds to the difference in maternal and paternal effect sizes. Only the five most significant eQTLs are listed

### Replication in SardiNIA

We attempted replication of our opposite effect GWAS and single parent association studies in the Sardinia population (Supplementary Table 5). Ten SNPs significantly associated with nine phenotypes (BMI, Total cholesterol, eosinophil count, triglycerides, FEV_1_, LDL-C, LVMI, neutrophil count, age of menarche) with opposite effects in the Hutterites were analyzed using the same method in the Sardinia population. The SNP on chromosome 5, rs77785972, associated with BMI was close to suggestively replicated in Sardinia with *p*-value 7.7e-05 using a replication *p*-value threshold of 0.005 to correct for the ten tests performed. However, the difference in parental effect size (−1.90) was in the opposite direction. The difference in effect size and *p*-value could be due to differences in sample sizes, transformations, or other standardization methods on the data or population differences. Of the remaining eight single parent associations with six phenotypes (SBP, age of menarche, LDL-C, Total cholesterol, CIMT, FEV_1_), only one SNP with FEV_1_ was nominally significant (*p*-value 0.0391; replication *p*-value threshold 0.003125 for 16 tests) in the Sardinians.

## Discussion

In this study, we introduced a statistical method that allows assessment of standard GWAS signals along with measures of differential POEs on common quantitative phenotypes. Similar to previous parent-of-origin studies of fewer phenotypes^6–8^, we tested for associations of maternally- or paternally derived alleles with each phenotype. We then extended this method to identify variants for which maternally- and paternally derived alleles have different, including opposite, effects on phenotypic values. Others have used similar methods to test for opposite effects in body weight and growth in mice^26^ or on methylation levels in humans^27^. In contrast, our study focused on 21 common disease-associated phenotypes in a single large pedigree and allowed us to broadly survey physiological effects of putative imprinted regions and the candidate genes at each associated locus.

Our studies of > 1000 Hutterites who are related to each other in a single pedigree allowed us to detect POEs, even when few genome-wide significant associations were detected in standard GWAS of the same phenotypes. Our method revealed parent-of-origin specific genome-wide significant associations for seven of the 21 phenotypes examined, with maternally inherited alleles associated with four phenotypes, paternally inherited alleles with three phenotypes (Table 1), and opposite parent-of-origin alleles with nine phenotypes, of which five also showed single POEs at different loci (Table 2). Overall, 11 of the 21 phenotypes examined showed genome-wide significant evidence of POEs with alleles at one or more loci. In contrast, standard GWAS of these same phenotypes and using the same markers in these same individuals revealed genome-wide significant association for only five traits.

It is notable that four of the nine significant opposite parent-of-origin effects (one each with LDL-C and triglycerides, and two with BMI) lie in or near long intergenic noncoding RNA genes (lincRNAs). LincRNAs are a feature of imprinted regions^1^, where they can silence the expression of genes on the opposite chromosome^3,28^. One of the variants, rs1032596, with an opposite parent-of-origin effect on LDL-C is located in the *LINC01081* gene. This noncoding RNA, along with *LINC01082*, regulates the *FOXF1* enhancer resulting in *FOXF1* parent- and tissue-specific activity^19^ providing experimental support for tissue-specific expression, a feature of imprinted regions.

Another variant with POEs in our study has been suggested to be imprinted in previously published work. The variant associated with opposite POEs for FEV_1_ is an eQTL for the gene *CNTN3*. *CNTN3* was shown to have exclusive maternal allele-specific expression in murine placentas^24^, although this finding may have been due to contaminating maternal cells^29,30^.

Other regions associated with POEs harbor genes involved in transcriptional repression (e.g., *SCMH1* with LVMI on chromosome 1) or the associated SNPs are reported as eQTLs in GTEx with expression in tissues relevant to the phenotype under investigation (e.g., the LVMI-associated SNPs are eQTLs for *XIRP2*, which is expressed in skeletal muscle and heart left ventricle)^18^. Overall, these patterns of expression provide additional support that the parent-of-origin associations in our study are flagging imprinted regions or regions involved in the regulation of gene expression. Finally, we used gene expression in LCLs from the Hutterites to directly test for parent-of-origin eQTLs among SNPs associated with phenotypes in the parent-of-origin GWAS. Although none of the parent-of-origin eQTLs met criteria for significance after correcting for multiple testing, the SNP on chromosome 9 with opposite POEs on total cholesterol levels was borderline significant as an opposite parent-of-origin eQTL for *POLR1E*, and possible for three other genes at the same locus (*PAX5*, *FBXO10*, and *FRMPD1*). The presence of multiple genes with potential parent-of-origin expression patterns is further supportive of an imprinted locus. The availability of gene expression only in LCLs from the Hutterites limits the inferences we can draw about effects on expression because imprinted regions are often tissue-specific and sometimes developmentally regulated^1,2^. Despite this limitation, the fact that many of the SNPs associated with POEs on phenotypes are themselves eQTLs in relevant tissues (GTEx) and some are suggestive of having POEs on expression in LCLs from the Hutterites is generally supportive of the suggestion that some of the regions identified in this study are imprinted or have network interactions with imprinted genes^31^ in humans. Additionally, our data suggest that loci with POEs influence a broad spectrum of quantitative phenotypes that are themselves risk factors for common diseases.

In particular, the discovery of POEs for eight traits that are associated cardiovascular disease risk is intriguing. These include metabolic phenotypes, such as BMI, total cholesterol, triglycerides, LDL, and age of menarche, that have indirect effects on cardiac health, as well as LVMI and CIMT, which more directly reflect cardiac health. Some of these phenotypes showed associations with paternally inherited alleles only (systolic blood pressure, LDL-C, total cholesterol), maternally inherited alleles only (LVMI, CIMT, and age at menarche), and/or with opposite effect variants (BMI, LDL-C, triglycerides, total cholesterol, LVMI, age at menarche). It has been suggested that genomic imprinting evolved in the mammalian lineage as a way to regulate maternally and paternally expressed genes in the placenta during pregnancy and modulate metabolic functions related to growth, where the parental interests may be in conflict—paternal alleles favoring growth of the fetus at the expense of the mother while maternal alleles favor restricting resources to the fetus to ensure preservation of her nutritional needs^3,28,32^. Our data show some effects that are consistent with this theory. For example, three independent paternally inherited alleles on chromosome 1 are associated with increased LDL-C (Fig. 2) and total cholesterol (Supplementary Fig. 7); a paternal allele on chromosome 13 is also associated with increased systolic blood pressure (Supplementary Fig. 6). However, it is not always possible to interpret our results in light of this model, such as the association of maternal allele on chromosome 2 with decreased CIMT (Supplementary Fig. 3), where decreased cardiovascular risk is associated with latter age of menarche, or the maternal allele on chromosome 16 associated with decreased age of menarche (Fig. 1), which is associated with increased cardiovascular risk^33^. However, because many of the traits associated with POEs in this study were measured in adults, and none were measured in neonates, we are likely observing the downstream effects of processes that occurred in utero. Nonetheless, this kinship theory, or parent-conflict hypothesis, could account for the enrichment of parent-of-origin associations, particularly those with opposite effects, among metabolic and CVD-associated traits^1^.

Although we identified two parent-of-origin associations with nominal significance in the Sardinia population, an opposite effect association with BMI and a single parent maternal association with FEV_1_, there are many potential reasons for the overall lack of replication. It is possible, for example, that the most significant SNP is not the causal SNP and LD structures differ in the Hutterites and the Sardinian population. Additionally, it is also possible that different genetic backgrounds modify these associations. Lastly, the Hutterites and Sardinians are exposed to different environments and have different lifestyles that could differentially affect the associations between genotype and phenotype.

Finally, we note that the parent-of-origin GWAS for 21 phenotypes in the Hutterites revealed overall twice as many genome-wide significant loci compared to standard GWAS of the same phenotypes in the same individuals, suggesting that variation at imprinted loci may represent some of the missing heritability of these phenotypes and potentially for the disease for which they confer risk. This idea is consistent with observations in both mice and humans^34^. POEs in mice contribute disproportionally to the heritability of 97 traits, including those related to total cholesterol, weight, HDL, and triglycerides^35^. Exactly how much loci with POEs in humans contribute to phenotypic variation and disease risk overall remains to be determined, but our study provides compelling evidence that it is likely to be significant for many important traits.

## Methods

### Sample composition

The individuals in this study have participated in one or more of our studies on the genetics of complex traits in the Hutterites^36–38^. The more than 1500 Hutterites in our study are related to each other in a 13-generation pedigree including 3671 individuals. Informed consent was obtained from all subjects, under University of Chicago IRB-approved protocols.

### Genotype data

Variants detected in the whole genome sequences of 98 Hutterites were previously imputed to an additional 1317 individuals who were genotyped on one of three Affymetrix arrays (500k, 5.0, and 6.0) using PRIMAL, a high-accuracy pedigree-based imputation method^17^. PRIMAL is a phasing and imputation software that uses pedigree-based identity-by-descent (IBD) information for accurate analyses. IBD segments are obtained with Hidden-Markov Models and organized into an IBD clique dictionary, a data structure for efficient lookup queries that enables fast imputation. IBD cliques serve the role of ‘parents’ in family-based phasing. The method is characterized by very high-accuracy on the chromosomes that are shared IBD. PRIMAL was used to phase alleles and assign parent-of-origin for 83% of about 12 million autosomal SNPs. For these studies, we selected SNPs that had a MAF > 1% and genotype call rate > 85%. This yielded 5,891,982 autosomal SNPs. Parent-of-origin allele call rates differed among individuals and between phenotypes (Supplementary Table 1).

### Phenotype data

We included 21 quantitative phenotypes that were previously measured in the Hutterites. Descriptions for each phenotype, as well as exclusion criteria, transformations, and covariates used with each phenotype in the GWAS, are available in the Supplementary Methods (Supplementary Table 1). Detailed descriptions for 18 of the 21 phenotypes can be found in Cusanovich et al.^36^ The remaining three are described here. Height was measured in cm on a stadiometer with shoes removed. BMI was calculated using weight (kg, measured on scale) divided by height (m) squared. Age at menarche was collected retrospectively by interview.

### Genome-wide association studies

We used a linear mixed model as implemented in GEMMA to test for genome-wide association with 21 phenotypes using an additive model. We corrected for relatedness, as well as relevant covariates (Supplementary Table 1). We used a threshold of significance at *p* < 5 × 10^–8^. This threshold does not account for the extensive linkage disequilibrium in the Hutterites (which reduces the effective number of tests) or for the 21 GWAS performed (which increases the number of tests). For these reasons, we consider the threshold to be anti-conservative. The results are summarized in Supplementary Table 2.

### Maternal and paternal GWAS

To evaluated the evidence for POEs, we tested maternal and paternal alleles separately with each phenotype, comparing phenotypic differences between the maternally inherited alleles and between the paternally inherited alleles. We used a linear mixed model as implemented in GEMMA, which allows us to correct for relatedness as a random effect, as well as sex, age, and other covariates as fixed effects^39^. The linear mixed model for the parent-of-origin GWAS for testing maternal alleles and paternal alleles is shown in Eqs. 1 and 2, respectively.

1 $$\begin{array}{*{20}{c}} {{\mathbf{Y}} = W\alpha + {\mathbf{X}}_{\mathbf{M}}{\boldsymbol{\beta }}_{\mathbf{M}} + g + \varepsilon } \end{array}$$

2 $$\begin{array}{*{20}{c}} {{\mathbf{Y}} = W\alpha + {\mathbf{X}}_{\mathbf{P}}{\boldsymbol{\beta }}_{\mathbf{P}} + g + \varepsilon } \end{array}$$

*n* is the number of individuals, **Y** is an *n* × 1 vector of quantitative traits, W is an *n* × *c* matrix of covariates (fixed effects) including intercept 1. **α** is a *c* × 1 vector of covariate coefficients. **X**_**M**_ is an *n* × 1 vector of maternal alleles, and **X**_**p**_ an *n* ×1 vector of paternal alleles. ***β***_**M**_ and ***β***_**p**_ are the effect sizes of maternal and paternal alleles, respectively. *g* is a vector of genetic effects with ${\mathbf{g}}\sim {\mathrm{N}}({0,{{A\sigma }}_{\mathrm{g}}^2})$where ***A*** is the genetic relatedness matrix; **ε** is a vector of non-genetic effects with ${\mathbf{\varepsilon }}\sim {\mathrm{N}}(0,{\mathrm{I\sigma }}_{\mathrm{e}}^2)$. Parent-of-origin allele frequency of significant SNPs are in Supplementary Table 6.

### Differential effect GWAS (PO-GWAS)

To test for a difference in the same allele inherited from each parent, including opposite effects, we re-parameterized the test model (Equation 3) from Garg et al.^8^ resulting in a regression model similar to that used in Wolf et al*.*^26,27^.The null model (Eq. 4) is a standard GWAS model, ignoring parent-of-origin of alleles. The test model (Eq. 3) is more significant when maternal and paternal alleles have differential effects on gene expression.

3 $$\begin{array}{*{20}{c}} {{\mathbf{Y}} = W\alpha + {\mathbf{X}}_{\mathbf{M}}{\boldsymbol{\beta }}_{\mathbf{M}} + {\mathbf{X}}_{\mathbf{P}}{\boldsymbol{\beta }}_{\mathbf{P}} + g + \varepsilon } \end{array}$$

4 $$\begin{array}{*{20}{c}} {{\mathbf{Y}} = W\alpha + {\mathbf{X}}_{{\mathbf{PM}}}{\boldsymbol{\beta }}_{{\mathbf{PM}}} + g + \varepsilon } \end{array}$$

This new model allows us to measure the difference in parental effect of the same allele when the genotype is a covariate in Eq. 5.

5 $$\begin{array}{*{20}{c}} {{\mathbf{Y}} = W\alpha + \frac{{\left( {{\mathbf{X}}_{\mathbf{M}} - {\mathbf{X}}_{\mathbf{P}}} \right)}}{2}\left( {{\boldsymbol{\beta }}_{\mathbf{M}} - {\boldsymbol{\beta }}_{\mathbf{P}}} \right) + {\mathbf{X}}_{{\mathbf{PM}}}\frac{{\left( {{\boldsymbol{\beta }}_{\mathbf{P}} + {\boldsymbol{\beta }}_{\mathbf{M}}} \right)}}{2} + g + \varepsilon } \end{array}$$

**X**_**PM**_ is a *n* × 1 vector of genotypes with possible values [0,1,2], equivalent to **X**_**p**_**+ X**_**M**_. $\left( {{\boldsymbol{\beta }}_{\mathbf{M}} - {\boldsymbol{\beta }}_{\mathbf{P}}} \right)$ is the difference in parental effect size. If the difference in parental effect size is large and significantly different from 0 it suggests a parent-of-origin effect exists at this variant. $\frac{{\left( {{\mathbf{X}}_{\mathbf{M}} - {\mathbf{X}}_{\mathbf{P}}} \right)}}{2}$ is a *n* × 1 vector of genotypes with possible values [−1,0,1]. $\frac{{({\boldsymbol{\beta }}_{\mathbf{P}} + {\boldsymbol{\beta }}_{\mathbf{M}})}}{2}$ is the average parental effect size that is captured in normal GWAS using genotypes The average genotypes are added in as a covariate, with the average parental effect size the corresponding covariate coefficient. This differential effect GWAS was tested in GEMMA using BIMBAM format to use average genotype values^40^. Parent-of-origin allele frequency of significant SNPs are in Supplementary Table 6.

### Parent-of-origin eQTL studies

RNA-seq data from LCLs were available for 430 Hutterites included in a previous study (50 bp single end reads; median depth of 10.5 million reads)^36^. For this study, sequencing reads were reprocessed as follows. Reads were trimmed for adaptors using Cutadapt (with reads < 5 bp discarded) then remapped to hg19 using STAR indexed with gencode version 19 gene annotations^41,42^. To remove mapping bias, reads were processed using WASP mapping pipeline^43^. Gene counts were collected using HTSeq-count^44^. VerifyBamID was used to identify sample swaps to include individuals that were previously excluded^45^. Genes mapping to the X and Y chromosome were removed; genes with a Counts Per Million (CPM) value of 1 (expressed with less than 20 counts in the sample with lowest sequencing depth) were also removed. Limma was used to normalize and convert counts to log transformed CPM values^46^. Technical covariates that showed a significant association at *p* < 0.05 with any of the top 10 principal components were regressed out (RNA Integrity Number and RNA concentration).

### Maternal and paternal parent-of-origin eQTL

LCL RNA-seq data was used to test the single parent model for the most significant SNP from the maternal or paternal only GWAS for each phenotype. We selected all genes detected as expressed in the LCLs and residing within 1 Mb of each most significant associated SNP. Summary of the SNPs and genes tested are in Supplementary Table 3.

### Differential parent-of-origin eQTL

LCL RNA-seq data was used to test the opposite effect model for the most significant SNP in each region that was associated with a phenotype in the parent-of-origin opposite effects GWAS. We selected all genes detected as expressed in the LCLs and residing within 1 Mb of each associated SNP. Summary of the SNPs and genes tested are in Supplementary Table 4.

### Replication in SardiNIA

The SardiNIA study is a longitudinal population-based cohort study started in 2001 to study quantitative traits of biomedical relevance with a special emphasis on those influencing aging. In a first survey, the project recruited 6148 individuals from four towns in the Lanusei Valley (east-central Sardinia) and assessed 98 quantitative traits in over 62% of the eligible population living in the region (age 14–102 years), and at least 96% of the initial cohort have all grandparents born in the same province. Recently, the study recruited 773 additional individuals, involving a total of 6921 subjects. The longitudinal study, now in its 14th year and in its fourth phase, collected the longitudinal information on more than 1000 quantitative traits, including inflammatory markers and immune related traits, that can be scored on a continuous scale. Written informed consent was obtained from all participants. Genotypes were obtained after imputation with a reference panel generated from low pass sequencing of 3514 Sardinians as described in Sidore et al.^47^. The parent-of-origin genotypes were evaluated with a custom script applied to the 1308 complete trios present in the cohort. Trait measures were adjusted by age and age squared; residuals were inverse-normalized by quantile transformation. Single parent effects were tested separately for maternal and paternal allele using epacts (test q.emmax)^48^. Opposite effects were tested with the same methods as in the Hutterites, described above.

### Code availability

Code for PO-GWAS: https://github.com/smozaffari/PO_GWAS

## Supplementary information

Description of Additional Supplementary Files

Supplementary Information

Supplementary Data 1

Supplementary Data 2

Supplementary Data 3

## Data Availability

The accession number for the Hutterite data reported in this paper are dbGaP:phs000185.
