# Supplementary material for: Parent-of-origin effects on quantitative phenotypes in a large Hutterite pedigree
Source: Commun Biol. 2019 Jan 18;2:28. doi: 10.1038/s42003-018-0267-4 (PMC6338666; doi:10.1038/s42003-018-0267-4)
Supplement: Supplementary file 1 — Description of Additional Supplementary Files [file 42003_2018_267_MOESM1_ESM.docx]

**Description of Additional Supplementary Files**

**File Name**: Supplementary Data 1

**Description**: Significant Maternal only associations. GWAS variants are not excluded (LDL chromosome 19). Including all SNPs in LD. This data was generated analyzed as part of the Maternal and Paternal GWAS.

**File Name**: Supplementary Data 2

**Description**: Significant Paternal only associations. GWAS variants are not excluded (LDL chromosome 19). Including all SNPs in LD. This data was generated analyzed as part of the Maternal and Paternal GWAS.

**File Name**: Supplementary Data 3

**Description**: Significant differential effect (or opposite effect) associations. GWAS variants are not excluded. Including all SNPs in LD. This data was generated analyzed as part of the GWAS for Differential Parent of Origin Effects.
