# Supplementary material for: Parent-of-origin effects on quantitative phenotypes in a large Hutterite pedigree
Source: Commun Biol. 2019 Jan 18;2:28. doi: 10.1038/s42003-018-0267-4 (PMC6338666; doi:10.1038/s42003-018-0267-4)
Supplement: Supplementary file 2 — Supplementary Information [file 42003_2018_267_MOESM2_ESM.pdf]

SUPPLEMENTARY FIGURES

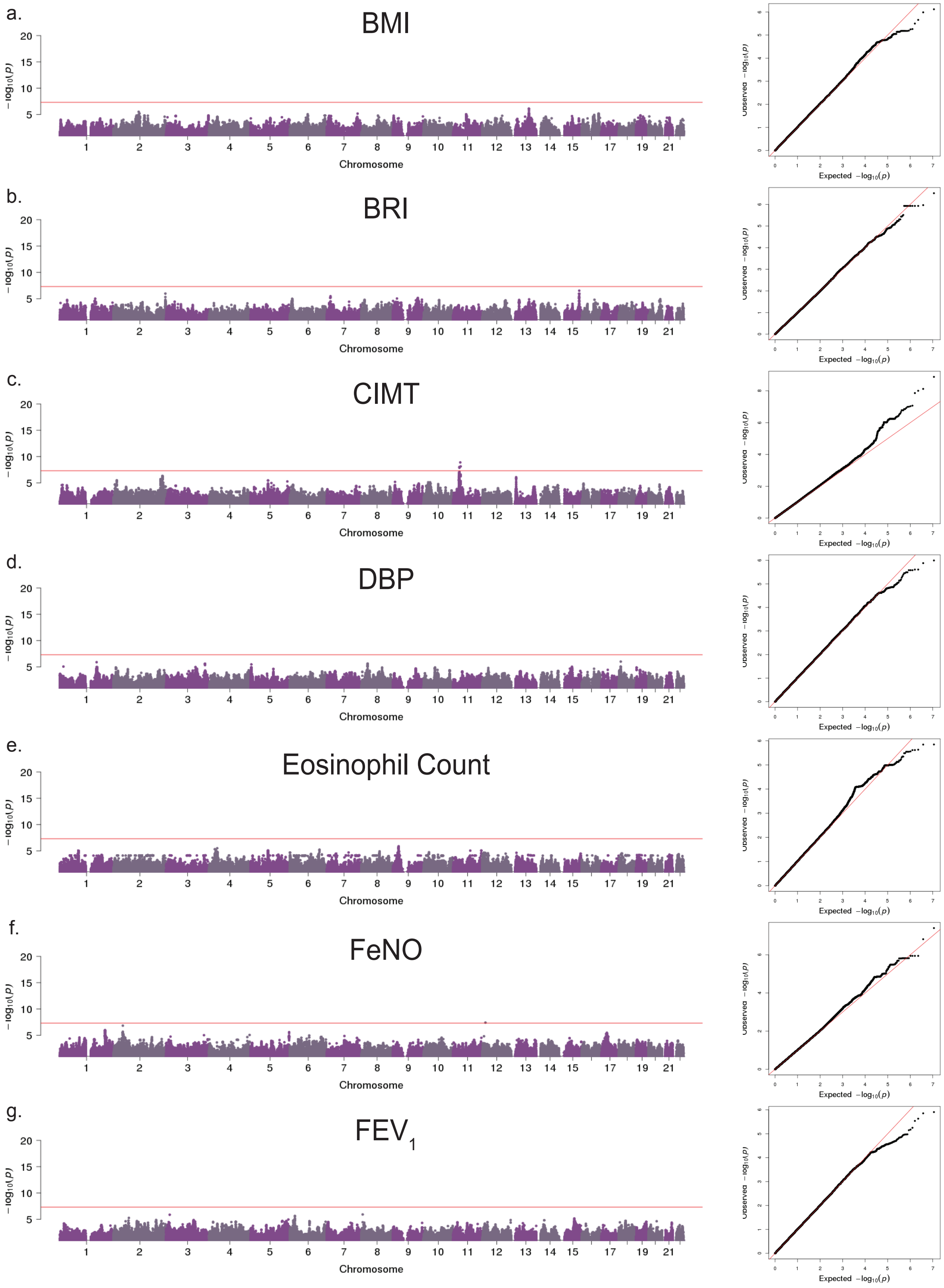

Supplementary Figure 1. Manhattan and QQ Plots from Standard GWAS of 21 Quantitative Phenotypes.

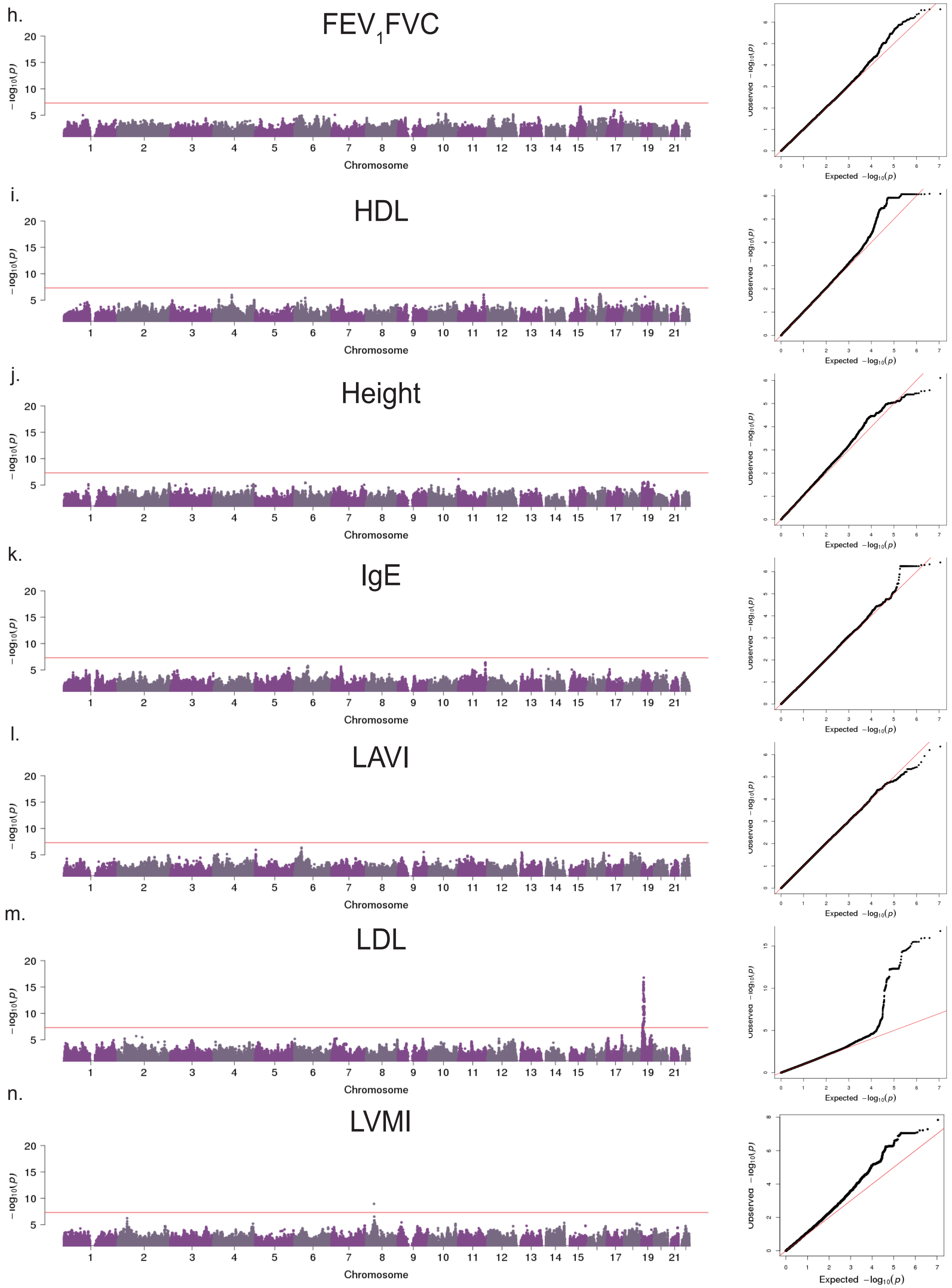

Supplementary Figure 1. (Continued) Manhattan and QQ Plots from Standard GWAS of 21 Quantitative Phenotypes.

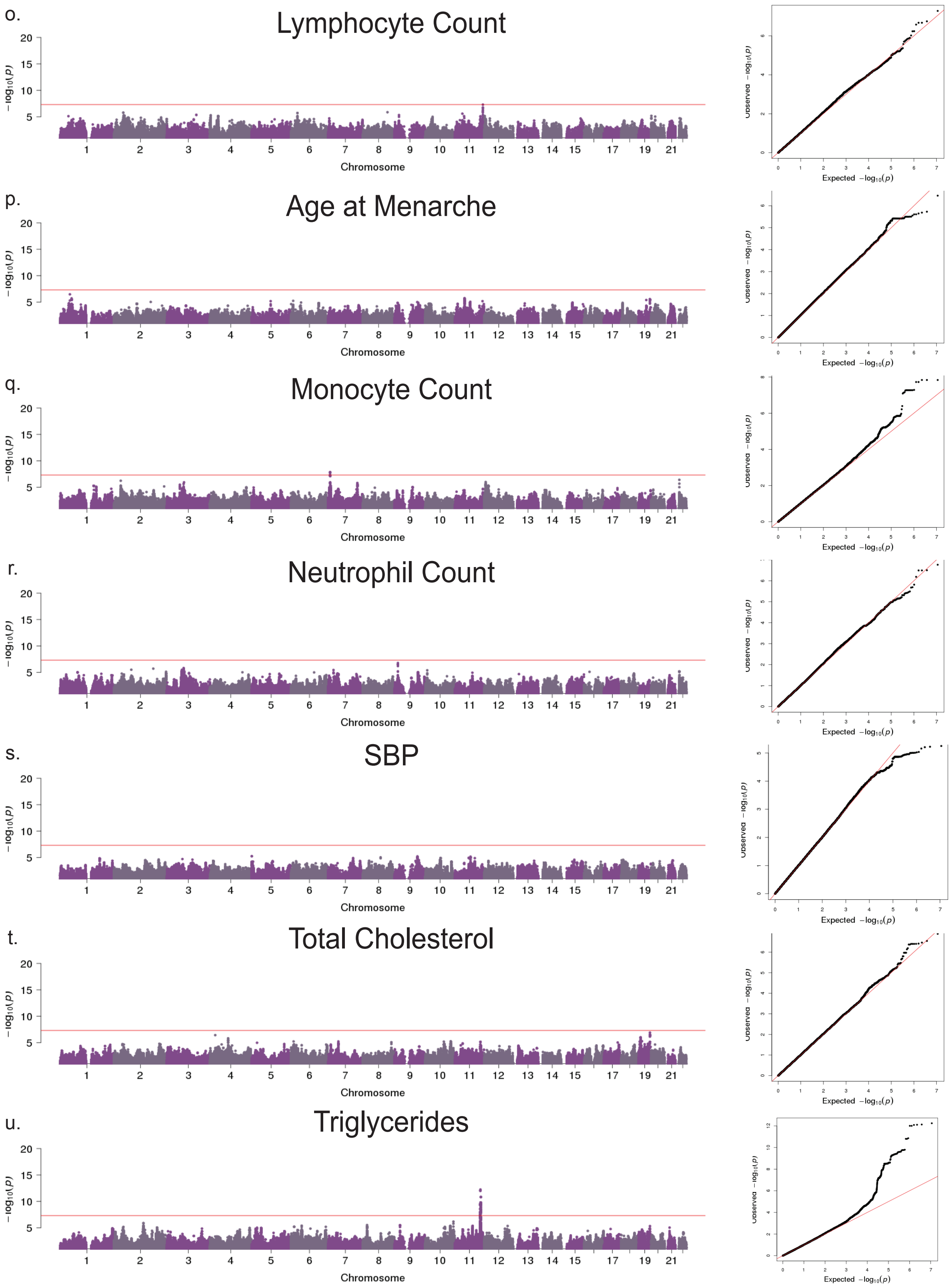

Supplementary Figure 1. (Continued) Manhattan and QQ Plots from Standard GWAS of 21 Quantitative Phenotypes.

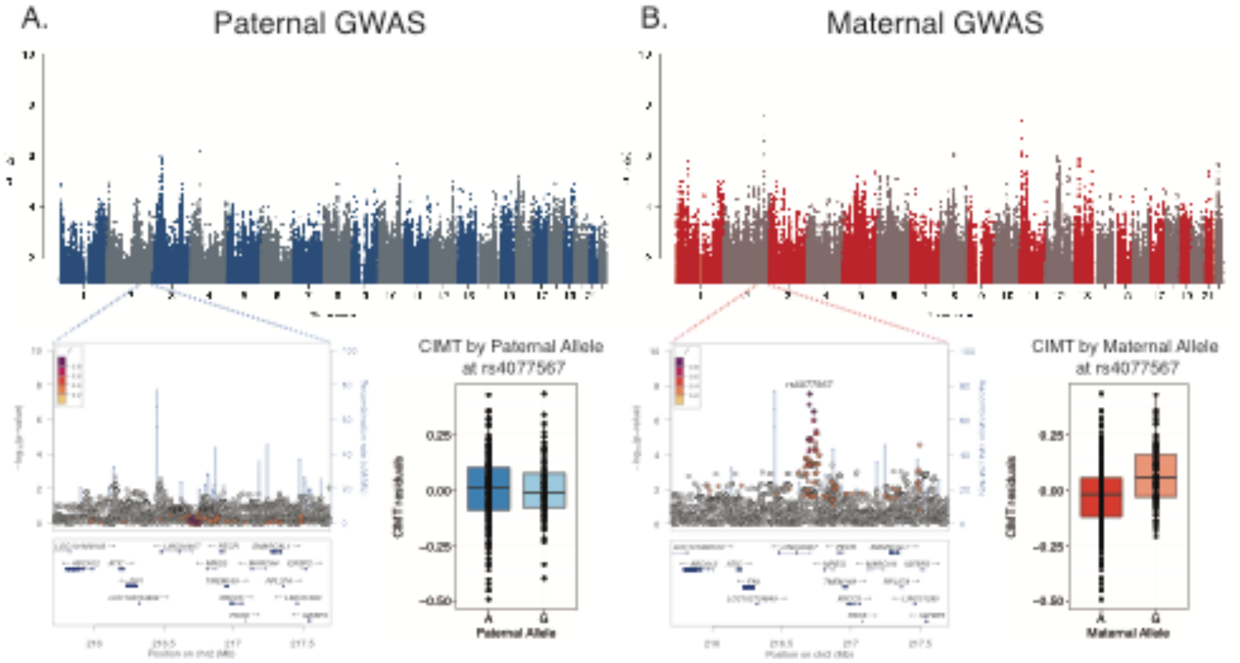

Supplementary Figure 2. Maternal and Paternal GWAS results for CIMT. The top panel shows the Manhattan plots from the paternal (A) and maternal (right) GWAS. LocusZoom plots are shown in the lower panel for the associated region in the GWAS. Boxplots show the distribution of CIMT residuals (the residuals correspond to the inverse of raw CIMT values) (y-axes) by the corresponding maternal and paternal alleles at this SNP (x-axes). The horizontal bar of the boxplot shows the median, the box delineates the first and third quartile, and the whiskers show +/-1.5 x IQR.

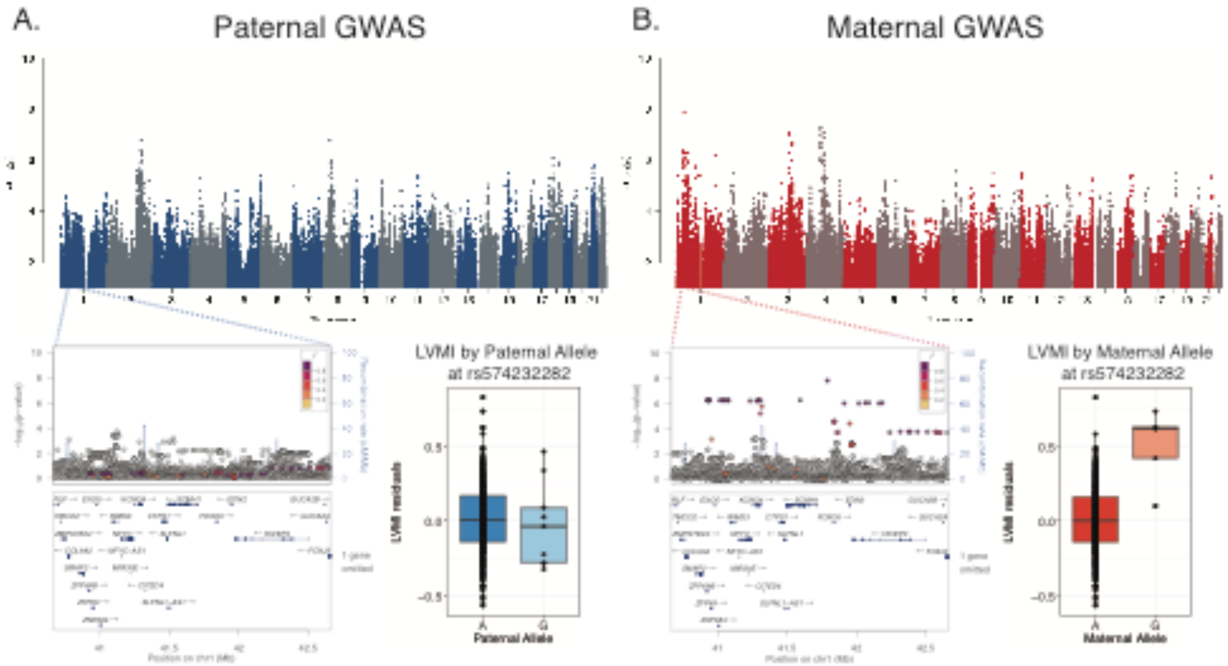

Supplementary Figure 3. Maternal and Paternal GWAS results for LVMI. The top panel shows the Manhattan plots from the paternal (A) and maternal (B) GWAS. LocusZoom plots for both GWAS are shown in the lower panel for the associated region in the GWAS. Boxplots show the distribution of LVMI residuals (y-axes) by the corresponding maternal and paternal alleles at this SNP (x-axes). The horizontal bar of the boxplot shows the median, the box delineates the first and third quartile, and the whiskers show +/-1.5 x IQR.

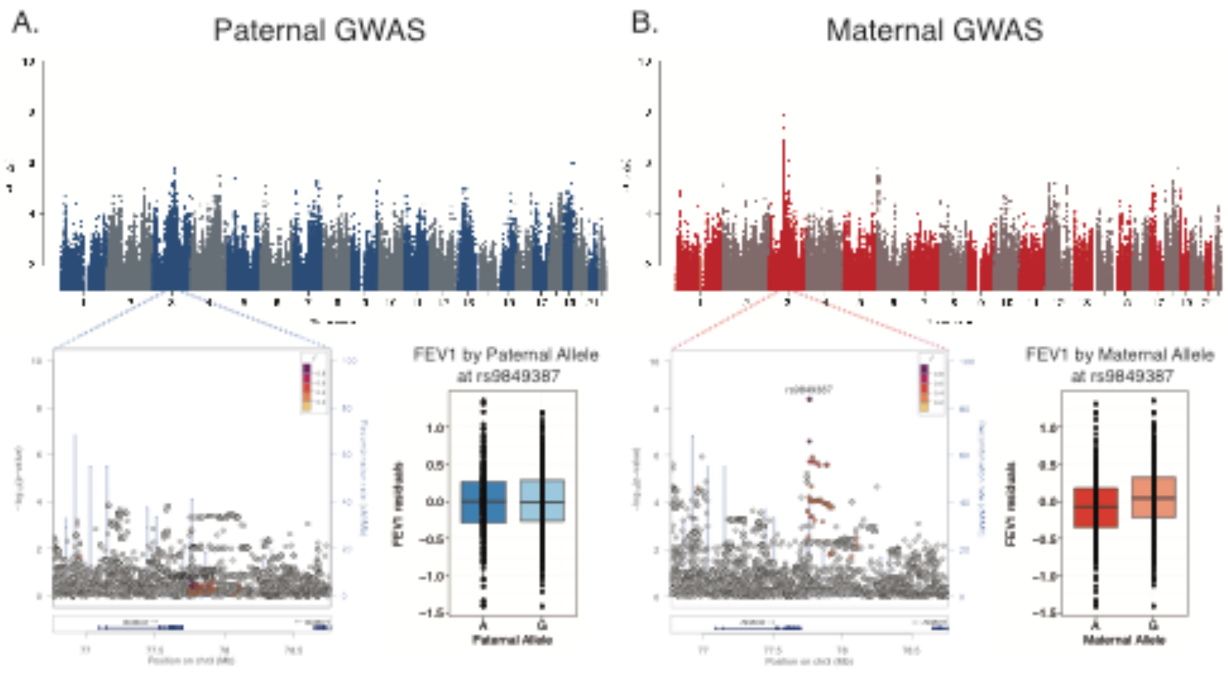

Supplementary Figure 4. Maternal and Paternal GWAS results for FEV$_1$. The top panel shows the Manhattan plots from the paternal (A) and maternal (right) GWAS. LocusZoom plots for both GWAS are shown in the lower panel for the associated region in the GWAS. Boxplots show the distribution of FEV$_1$ residuals (y-axes) by the corresponding maternal and paternal alleles at this SNP (x-axes). The horizontal bar of the boxplot shows the median, the box delineates the first and third quartile, and the whiskers show +/-1.5 x IQR.

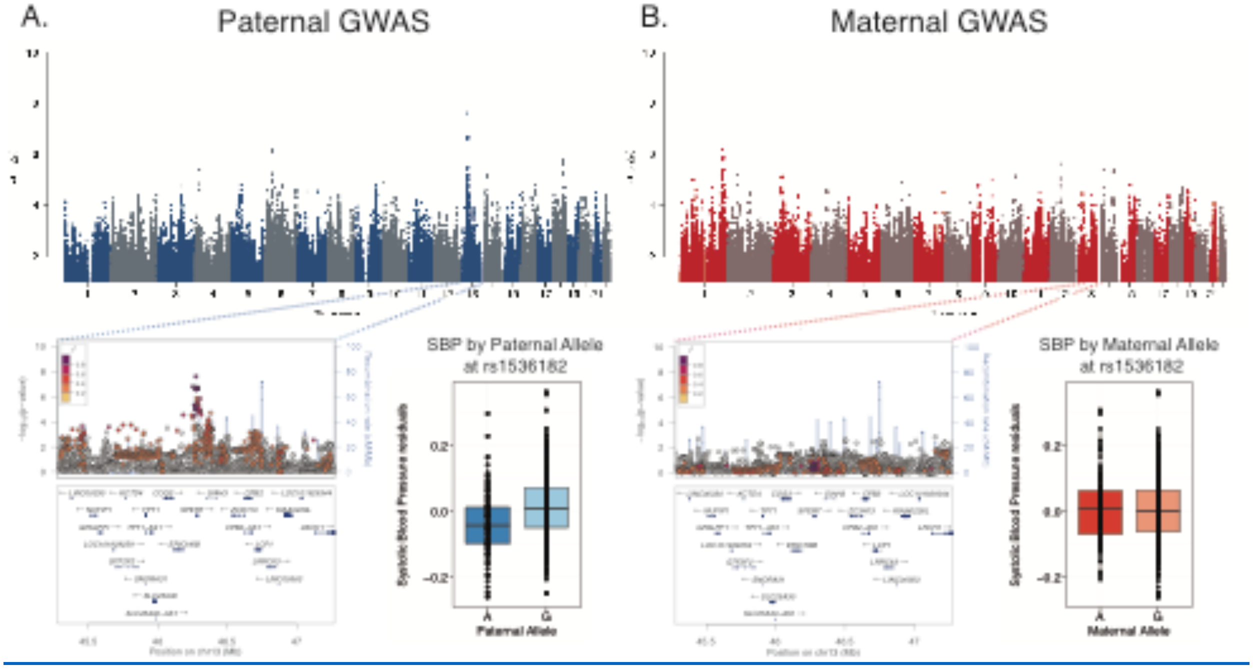

Supplementary Figure 5. Maternal and Paternal GWAS results for Systolic Blood Pressure. The top panel shows the Manhattan plots from the paternal (A) and maternal (right) GWAS. LocusZoom plots for both GWAS are shown in the lower panel for the associated region in the GWAS. Boxplots show the distribution of systolic blood pressure residuals (y-axes) by the corresponding maternal and paternal alleles at this SNP (x-axes). The horizontal bar of the boxplot shows the median, the box delineates the first and third quartile, and the whiskers show +/-1.5 x IQR.

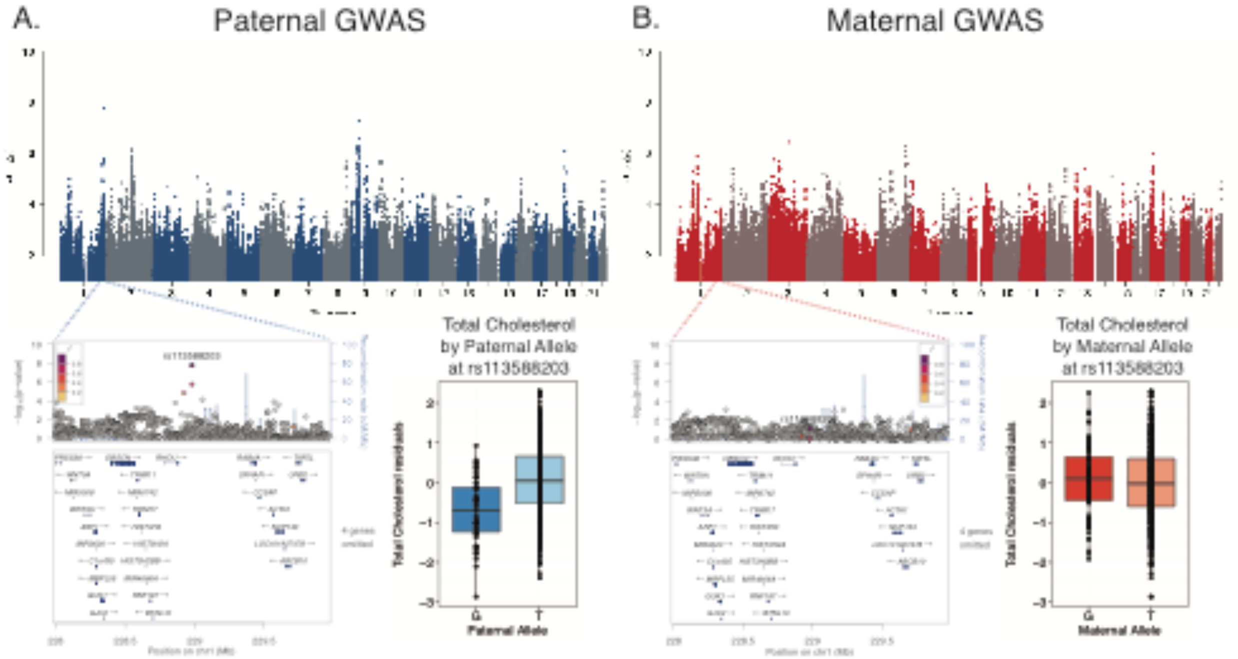

Supplementary Figure 6. Maternal and Paternal GWAS results for Total Cholesterol. The top panel shows the Manhattan plots from the paternal (A) and maternal (right) GWAS. LocusZoom plots for both GWAS are shown in the lower panel for the associated region in the GWAS. Boxplots show the distribution of total cholesterol residuals (y-axes) by the corresponding maternal and paternal alleles at this SNP (x-axes). The horizontal bar of the boxplot shows the median, the box delineates the first and third quartile, and the whiskers show +/-1.5 x IQR.

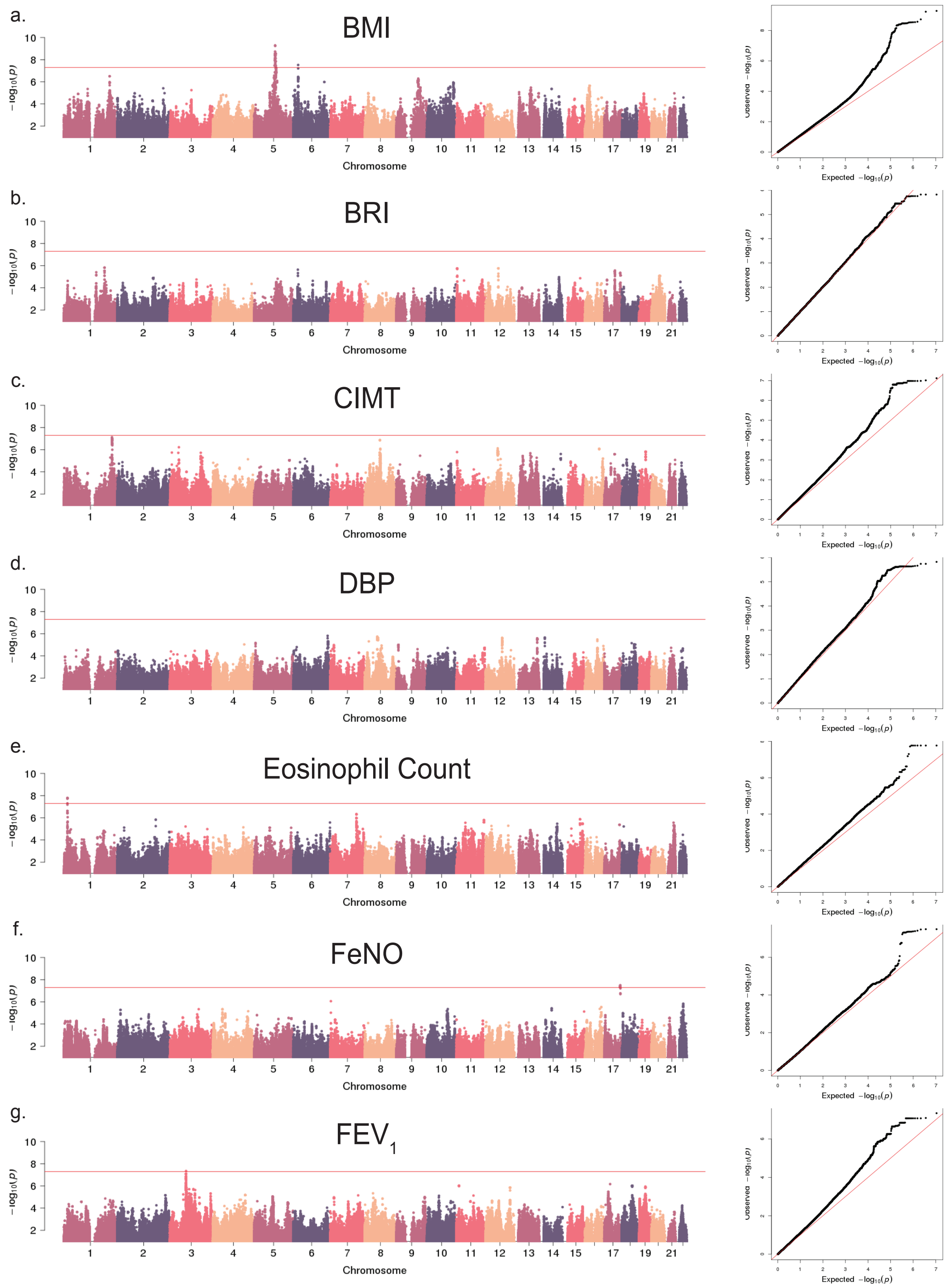

Supplementary Figure 7. Manhattan and QQ Plots from Differential Effect GWAS of 21 Quantitative Phenotypes.

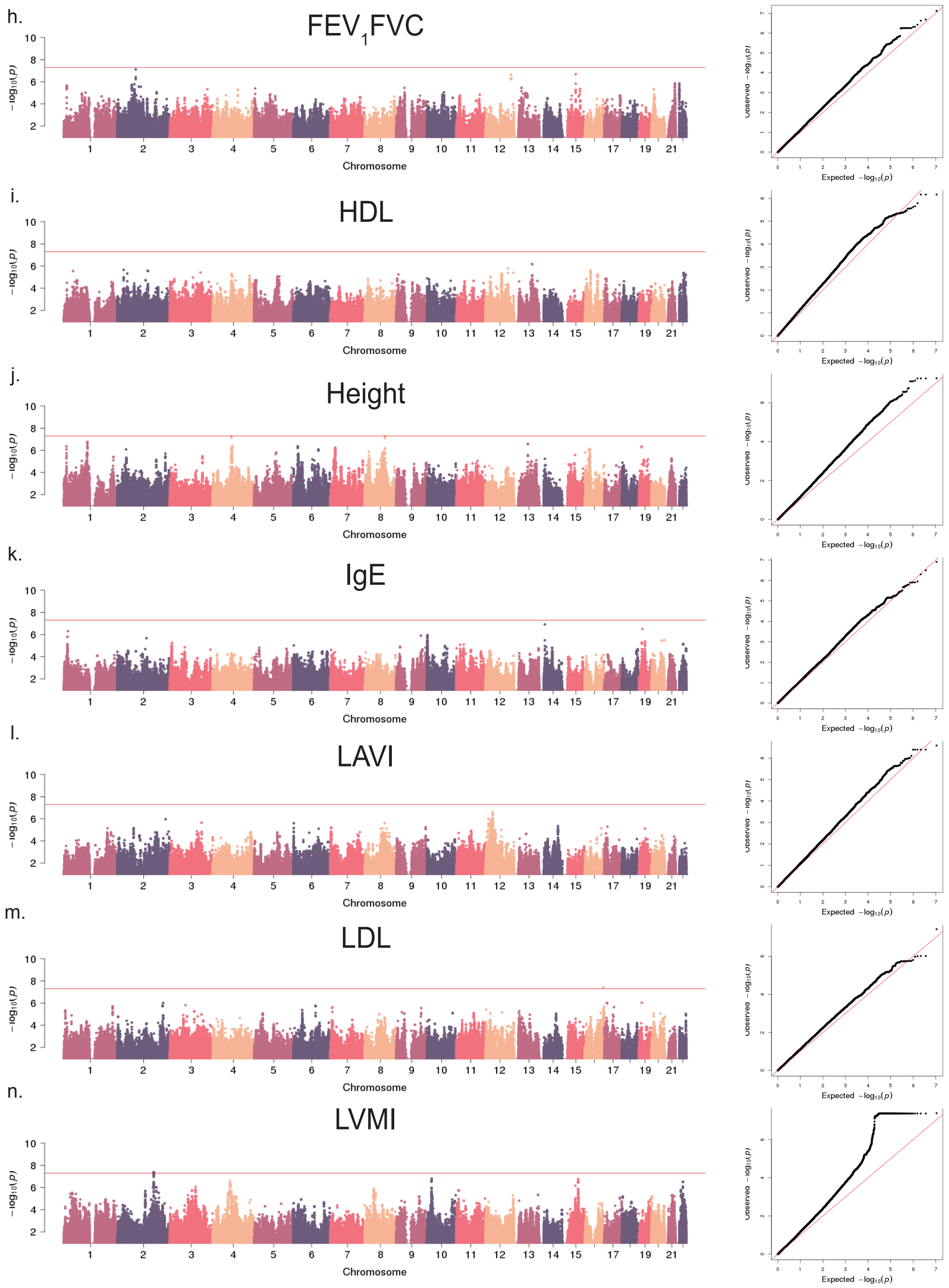

Supplementary Figure 7. (Continued) Manhattan and QQ Plots from Differential Effect GWAS of 21 Quantitative Phenotypes.

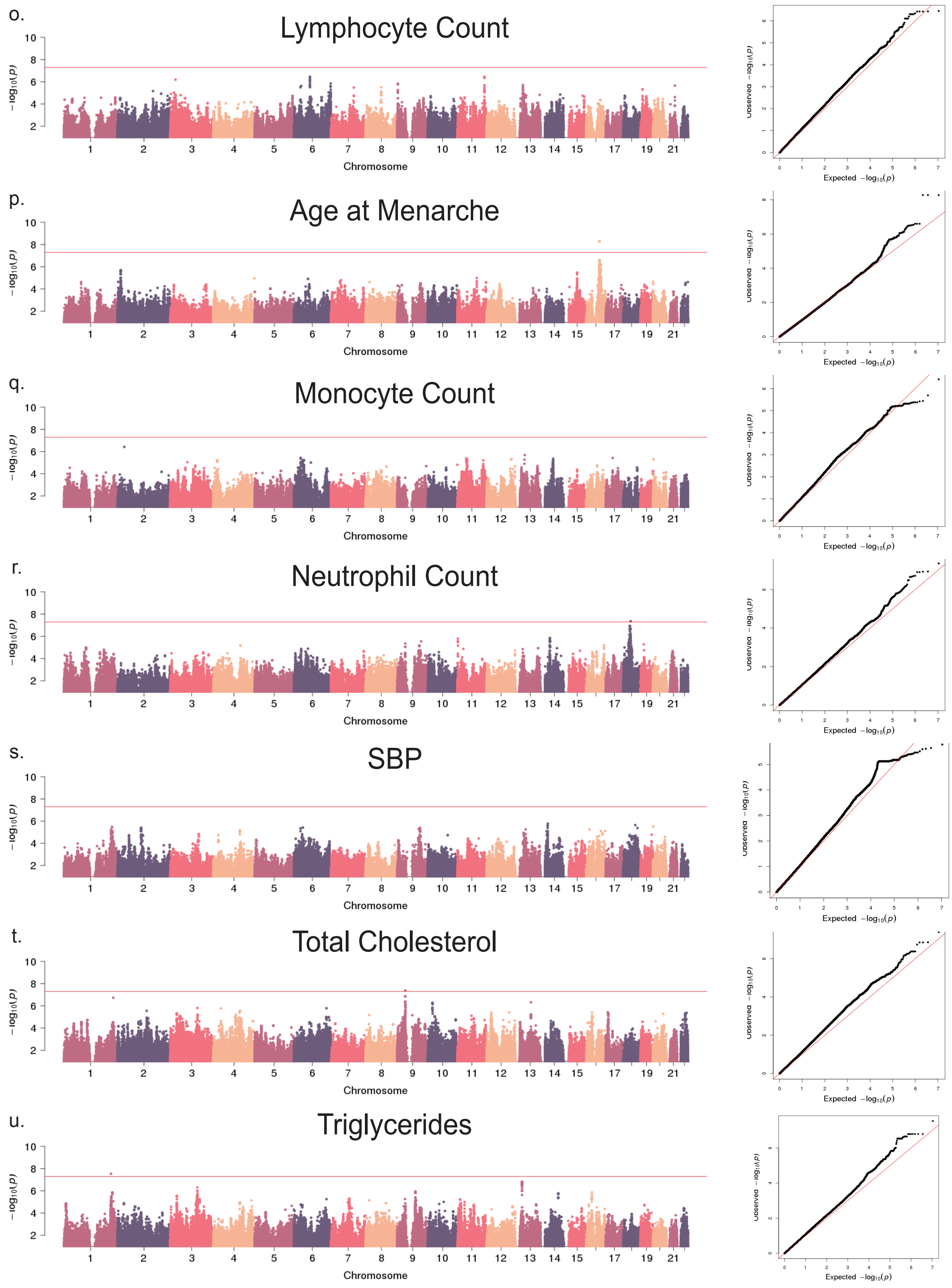

Supplementary Figure 7. (Continued) Manhattan and QQ Plots from Differential Effect GWAS of 21 Quantitative Phenotypes.

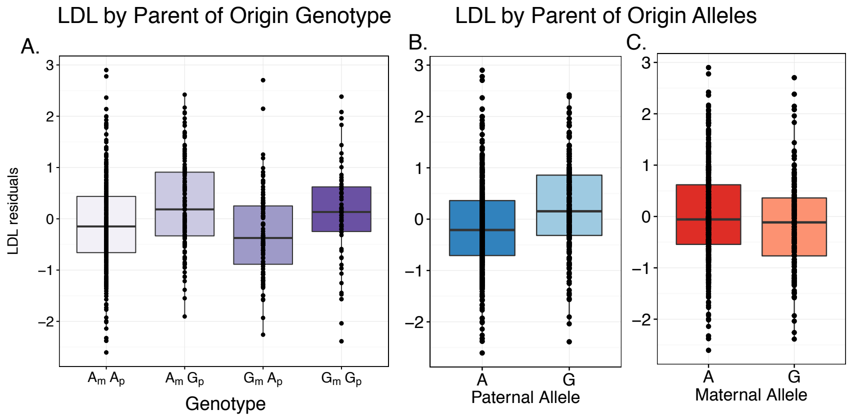

rs1032596 (chr16)

Supplementary Figure 8. Opposite Effect Parent of Origin GWAS Result for LDL. Box plots of LDL residuals (y-axes) are shown for each of the four genotypes (A), and for paternal (center panel; x-axis) and maternal (B) alleles. The maternal C allele is associated with decreased and maternal T allele with increased LDL. The paternal C allele is associated with increased and the paternal T allele with decreased LDL. The horizontal bar of the boxplot shows the median, the box delineates the first and third quartile, and the whiskers show +/-1.5 x IQR.

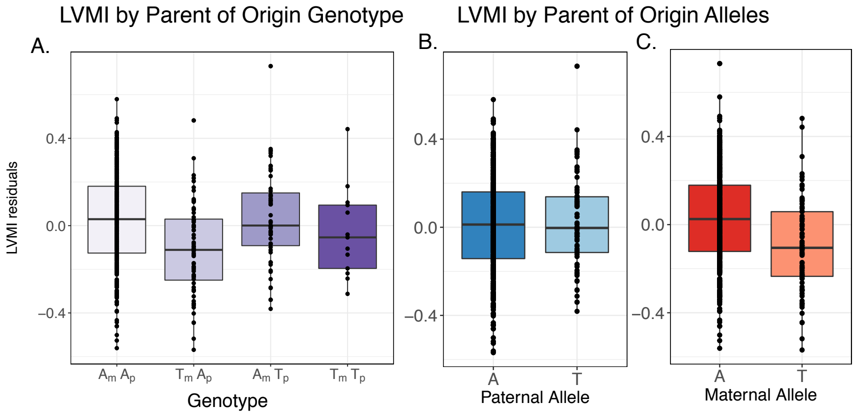

rs16853098 (chr2)

Supplementary Figure 9. Opposite Effect Parent of Origin GWAS Result for LVMI. Box plots of LVMI residuals (y-axes) are shown for each of the four genotypes (A), and for paternal (center panel; x-axis) and maternal (B) alleles. The maternal C allele is associated with decreased and maternal T allele with increased LVMI. The paternal C allele is associated with increased and the paternal T allele with decreased LVMI. The horizontal bar of the boxplot shows the median, the box delineates the first and third quartile, and the whiskers show +/-1.5 x IQR.

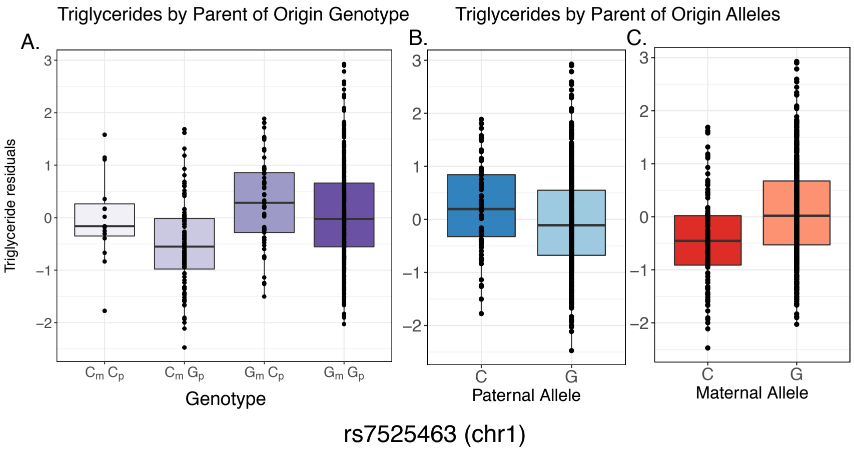

Supplementary Figure 10. Opposite Effect Parent of Origin GWAS Result for Triglycerides. Box plots of triglyceride residuals (y-axes) are shown for each of the four genotypes (A), and for paternal (center panel; x-axis) and maternal (B) alleles. The maternal C allele is associated with decreased and maternal T allele with increased triglycerides. The paternal C allele is associated with increased and the paternal T allele with decreased triglycerides. The horizontal bar of the boxplot shows the median, the box delineates the first and third quartile, and the whiskers show +/-1.5 x IQR.

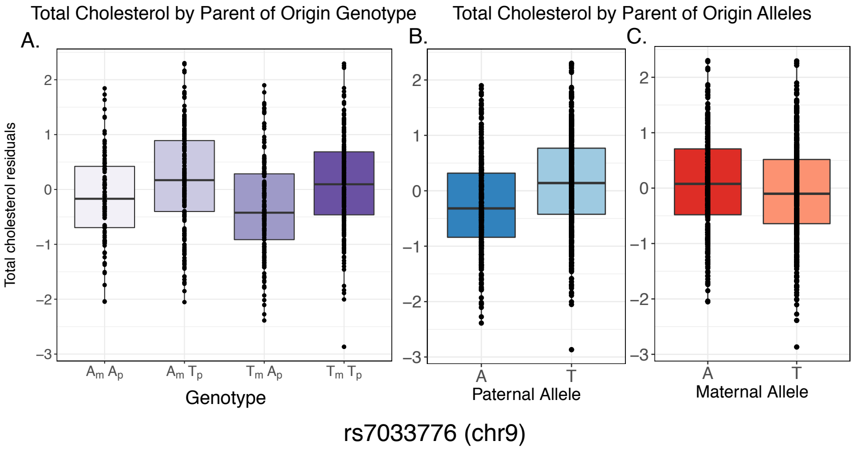

Supplementary Figure 11. Opposite Effect Parent of Origin GWAS Result for Total Cholesterol. Box plots of total cholesterol residuals (y-axes) are shown for each of the four genotypes (A), and for paternal (center panel; x-axis) and maternal (B) alleles. The maternal C allele is associated with decreased and maternal T allele with increased total cholesterol. The paternal C allele is associated with increased and the paternal T allele with decreased total cholesterol. The horizontal bar of the boxplot shows the median, the box delineates the first and third quartile, and the whiskers show +/-1.5 x IQR.

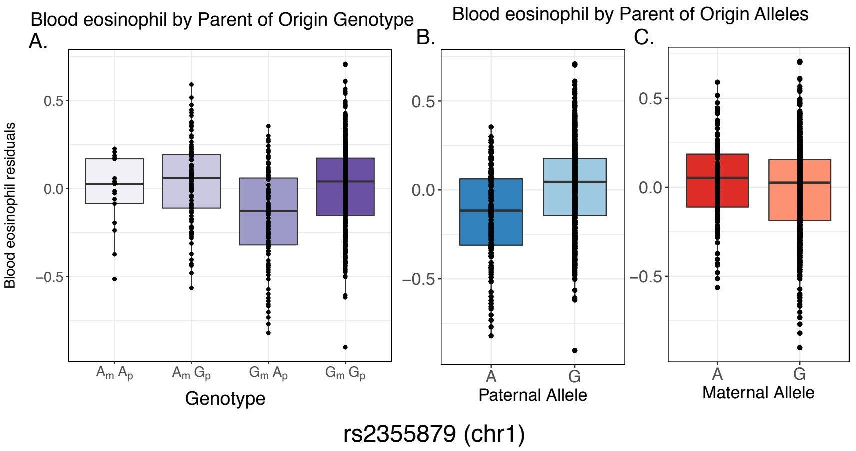

Supplementary Figure 12. Opposite Effect Parent of Origin GWAS Result for Blood Eosinophil Count. Box plots of eosinophil residuals (y-axes) are shown for each of the four genotypes (A), and for paternal (center panel; x-axis) and maternal (B) alleles. The maternal C allele is associated with decreased and maternal T allele with increased eosinophil count. The paternal C allele is associated with increased and the paternal T allele with decreased eosinophil count. The horizontal bar of the boxplot shows the median, the box delineates the first and third quartile, and the whiskers show +/-1.5 x IQR.

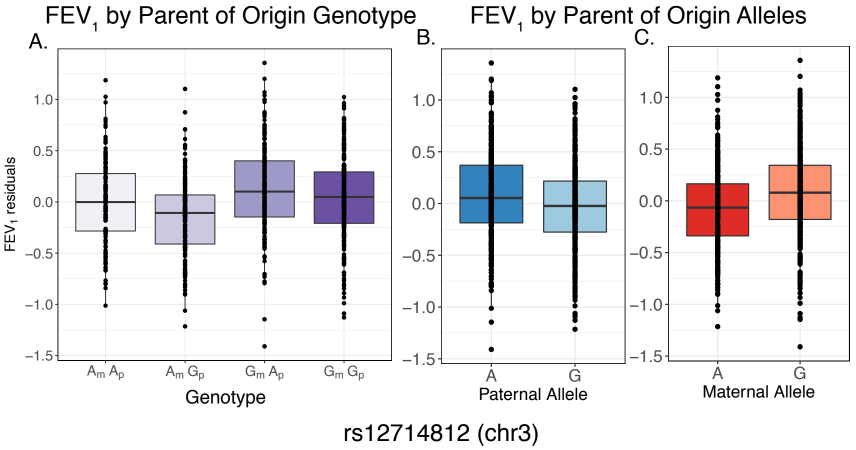

Supplementary Figure 13. Opposite Effect Parent of Origin GWAS Result for FEV_1. Box plots of FEV_1 residuals (y-axes) are shown for each of the four genotypes (A), and for paternal (center panel; x-axis) and maternal (B) alleles. The maternal C allele is associated with decreased and maternal T allele with increased FEV_1. The paternal C allele is associated with increased and the paternal T allele with decreased FEV_1. The horizontal bar of the boxplot shows the median, the box delineates the first and third quartile, and the whiskers show +/-1.5 x IQR.

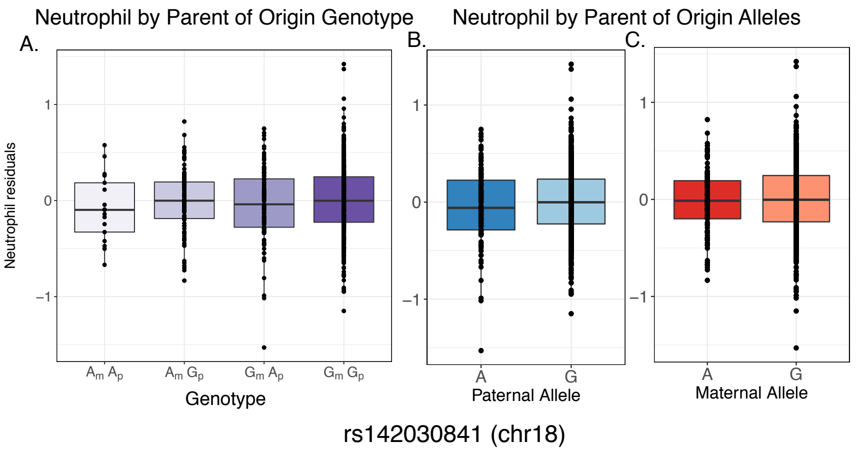

Supplementary Figure 14. Opposite Effect Parent of Origin GWAS Result for Neutrophil Count. Box plots of neutrophil residuals (y-axes) are shown for each of the four genotypes (A), and for paternal (center panel; x-axis) and maternal (B) alleles. The maternal C allele is associated with decreased and maternal T allele with increased neutrophil count. The paternal C allele is associated with increased and the paternal T allele with decreased neutrophil count. The horizontal bar of the boxplot shows the median, the box delineates the first and third quartile, and the whiskers show +/-1.5 x IQR.

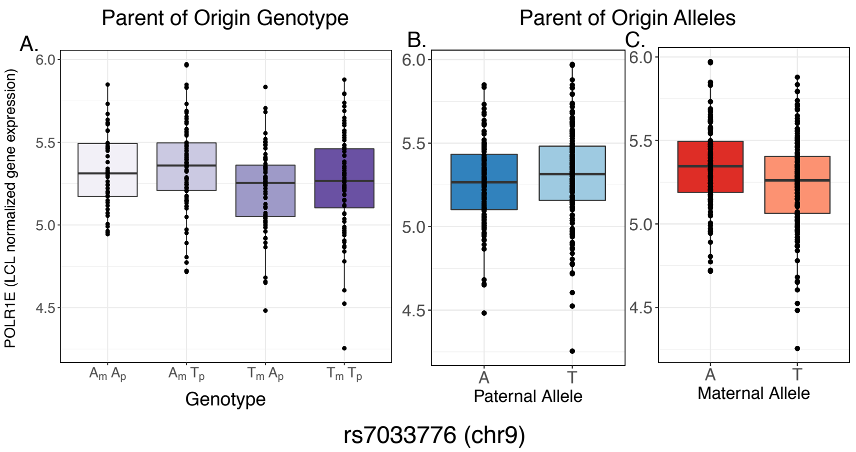

Supplementary Figure 15. Opposite Effect eQTL for rs7033776. Box plots of two significant loci plot *POLR1E* gene expression residuals (y-axes) for each of the four genotypes (A), and for paternal (center panel; x-axis) and maternal (B) alleles. The maternal T allele is associated with decreased and maternal A allele with increased *POLR1E* expression. The paternal T allele is associated with increased and the paternal A allele with decreased *POLR1E* expression. The horizontal bar of the boxplot shows the median, the box delineates the first and third quartile, and the whiskers show +/-1.5 x IQR.

SUPPLEMENTARY TABLES

Quantitative Trait	GWAS Sample Size	Sex Ratio (M/F)	Age ranges (years)	GWAS call rate	PO call rate	Transformation	Covariates	Exclusions
SBP	807	371/436	6-85	0.94	0.82	log	age, sex, age*sex, technician	Anti-hypertensive medication
DBP	807	371/436	6-85	0.94	0.82	log	age, sex, age*sex, inbreeding, technician	
HDL	828	381/447	14-85	0.94	0.80	cube root	age, sex	Anti-hypercholesterolemia medication, hormone replacement therapy, birth control; diagnosis of sitosterolemia
LDL	807	367/440	14-85		0.80	cube root	age, sex	
Triglycerides	828	382/446	14-85	0.94	0.80	log	age, sex	
Total cholesterol/HDL	828	381/447	14-85	0.94	0.80	log	age, sex	
Monocyte count	1069	503/566	5.47-85.10	0.94	0.81	log	age, sex, age*sex	Antibiotics, immunosupressants, and/or steroids was an exclusion for all WBC count phenotypes. Antifungal medication was an exclusion for the Eosinophil Count phenotype.
Lymphocyte count	1079	507/572	6-85	0.94		log	age	
Eosinophil count	1068	502/566	5.47-85.10	0.94	0.81	square root(log())	sex	
Neutrophil count	1070	503/567	5.47-85.10	0.94	0.81	log	age, sex, age*sex, phase	
LAVI	637	296/341	14-88	0.94	0.80	log	age, sex	Pregnant, history of heart valve replacement, or poor quality echocardiography images
LVMI	621	286/335	14-88	0.94	0.79	log	age, sex	Aortic stenosis by history or echocardiogram Cholesterol and/or thyroid medication or poor quality echocardiography images
CIMT	547	248/299	14-86	0.94	0.80	inverse	age, sex	Cholesterol and/or thyroid medication, history of liver disease, or poor quality imaging
FeNO	825	381/444	6-85	0.95	0.82	log	age, sex, technician	Poor quality measurements
BRI	950	445/505	6.11-78.22	0.94	0.82	none	age, inbreeding	Pregnant and/or lactating or medical contraindications (e.g. beta-blockers, asthma medication 24 hour prior to examination, cystic fibrosis)
FEV1	1102	509/593	5.47-8.47	0.94	0.82	none	Age, sex, height, height squared, phase, sex * age group (<17 vs ≥17 years old), age*sex*age group, height*sex*age group	Poor quality FEV1 and/or FVC measurements or medical contraindications (e.g. asthma medication 24 hour prior to examination, cystic fibrosis)
FEV1/FVC	1106	512/594	5.47-8.47	0.94	0.82	none	age, sex, age*sex, inbreeding	
Total serum IgE	1219	562/657	6-91	0.94	0.81	log	age	Xolair medication
BMI	1188	577/652	5.5-89.2	0.94	0.82	log	log(age), sex, phase, colony	Pregnancy
Height	1199	576/669	5-89	0.94	0.82	none	age, age*sex group (<15 years old males+females), age*sex (≥15 years old males), age*sex ≥15 years old females)	
Age at menarche	477	0/719	9-17	0.92	0.71	none	birthyear	

Supplementary Table 1. Summary of the Hutterite Phenotypes and Sample Composition. SBP stands for Systolic Blood Pressure, DBP: Diastolic Blood Pressure, HDL: High Density Lipoprotein, LDL: Low Density Lipoprotein, LAVI: Left Atrial Volume Index, LVMI: Left Ventricular Mass Index, CIMT: Carotid Intima Media Thickness, FeNO: Fraction of Exhaled Nitric Oxide, FEV_1: Forced Expiratory Volume at 1 s, FVC: Forced Vital Capacity, BRI: Bronchial Responsiveness Index

Phenotype	GWAS sample size	Most significant GWAS p-value	SNP Sample Size	rsid (Effect allele/ Other allele)	chr:loc	Beta (SE)	Variant	(Nearest) Gene	MAF
BMI	1188	8.04E-07	1016	rs139659764 (A/G)	13:81274241	-8.16E-02 (1.64E-02)	intergenic	*LINC00377*	0.07555
BRI	950	3.05E-07	935	rs7498042 (T/G)	15:91119014	3.34E-02 (6.47E-03)	intron	*CRTC3*	0.2921
CIMT	547	1.51E-09	470	rs116908536 (A/G)	11:33699981	-0.144 (2.34E-02)	intron	*LOC105376617*	0.05183
DBP	807	9.66E-07	999	rs116196949 (T/C)	18:9014768	-4.84E-02 (9.82E-03)	intergenic	*NDUFV2*	0.1312
Eosinophil count	1068	1.44E-07	939	rs62544465 (A/G)	9:26045558	8.17E-02 (1.68E-02)	intergenic	*LOC100506422*	0.147
FeNO	825	2.15E-07	753	rs1121780 (T/C)	2:42961321	0.157 (2.96E-02)	intron	*MTA3*	0.4824
FEV_1	1102	1.22E-06	1102	rs13281444 (C/T)	8:6520628	1.11E-01 (2.28E-02)	intron	*LOC100507530*	0.358
FEV_1/FVC	1106	2.49E-07	1099	rs8028898 (A/G)	15:71679543	-2.75E-01 (5.31E-02)	intron	*THSD4*	0.2049
HDL	828	8.81E-07	828	rs186133312 (A/T)	16:58850424	-0.483 (9.73E-02)	intergenic	*GOT2*	0.0687
Height	1199	7.62E-07	1009	rs1318252 (G/A)	11:1653200	1.38 (0.278)	intergenic	*KRTAP5-5*	0.4239
IgE	1219	3.99E-07	1187	rs66498879 (C/T)	11:125495211	-0.256 (5.045E-02)	5' UTR	*CHEK1*	0.3243
LAVI	637	3.07E-06	573	rs17205373 (G/C)	6:32626210	-0.1144 (2.81E-02)	intergenic	*HLA-DQB1*	0.09724
LDL	807	2.83E-17	714	rs557778817 (T/C)	19:11305534	1.3 (0.15)	intron	*KANK2*	0.03382
LVMI	621	1.23E-09	517	rs113389683 (A/G)	8:36026788	0.153 (2.47E-02)	intergenic	*UNC5D, KCNU1*	0.08624
Lymphocyte count	1079	5.67E-08	894	rs4323874 (G/A)	11:129110347	8.19E-02 (1.49E-02)	intergenic	*ARHGAP32*	0.4395
Age at Menarche	477	3.17E-07	463	rs785474 (G/C)	1:46609106	4.5E-01 (8.72E-02)	intron	*PIK3R3*	0.4268
Monocyte count	1069	1.36E-08	902	rs531317434 (G/T)	7:9466593	-0.27 (4.73E-02)	intergenic	*NXPH1, PER4*	0.03361
Neutrophil count	1070	2.1E-07	1070	rs1215134 (C/T)	9:15347518	-8.97E-02 (1.71E-02)	intergenic	*TTC39B*	0.4307
SBP	807	5.23E-06	774	rs28742608 (G/T)	5:1440934	-4.8E-02 (1.05E-02)	intron	*SLC6A3*	0.07549
Total Cholesterol	828	1.20E-07	828	rs11084211 (A/G)	19:53460861	0.287 (5.39E-02)	intron	*ZNF816, ZNF816-ZNF321P*	0.3071
Triglycerides	828	7.12E-13	707	rs184333869 (T/C)	11:117947268	-1.28 (0.175)	intron	*TMPRSS4*	0.02399

Supplementary Table 2. GWAS Results. Phenotype definitions, exclusions, transformations and covariates are summarized in **Supplementary Table 1**.

Phenotype	SNP	Genes +/- 1Mb expressed in LCLs
A. Maternal		
Age at Menarche	rs7184983	*AMFR, ARL2BP, BBS2, CCDC102A, CCL17, CCL22, CES5A, CETP, CIAPIN1, COQ9, CPNE2, CX2CL1, DOK4, FAM192A, GNAO1, HERPUD1, MR1E, MT1L, MT2A, NLRC5, NUDT21, NUP93, OGFOD1, PLLP, POLR2C, RSPRY1, SLC12A3*
CIMT	rs4077567	*ABCA12, ATIC, FN1, IGFBP2, MREG, PECR, RPL37A, SMARCAL1, TMEM169, XRCC5*
FEV_1	rs9849387	*ROBO1*
	rs6791779	*ZNF717*
LVMI	rs574232282	*CITED4, COL9A2, FOXJ3, HIVEP3, KCNQ4, NFYC, RIMS3, RLF, SCMH1, SMAP2, ZMPSTE24, SNF684*
B. Paternal		
LDL	rs12024326	*ADAMTS10, ANGPTL4, AHGEF18, CAMSAP3, CCL25, CD209, CD320, CERS4, CLEC4G, CTXN1, ELAVL1, EMR4P, EVI5L, FCER2, HNRNPM, INSR, KANK3, MAP2K7, MARCH2, MCOLN1, MYO1F, NDUFA, PCP2, PEX11G, PNPLA6, RAB11B, RAB11B-AS1, RPS28, SNAPC2, STXBP2, TIMM44, TRAPPC5, XAB2, ZNF358, ZNF414, ZNF557, ZNF558*
	rs4843650	*BANP, C16orf95, FBXO31, KLHDC4, MAP1LC3B, SLC7A5, ZC3H18, ZCCHC14, ZFPM1, ZNF469*
SBP	rs1536182	*COG3, CTF2F2, KIAA0226L, LCP1, LRCH1, NUFIP1, SLC25A30, SLC25A30-AS1, TPT1, TPT1-AS1, ZC3H13*
Total Cholesterol	rs113588203	*ABCB10, ARF1, C1orf35, GUK1, HIST3H2A, IBA57, IBA57-AS1, MRPL55, NUP133, OBSCN, RAB4A, RHOU, RNF187, SNORA51, SPHAR, TAF5L, TRIM11, TRIM17, URB2, WNT3A*

Supplementary Table 3. Candidate Genes for Parent of Origin eQTL.

Phenotype	SNP	Genes +/- 1Mb expressed in LCLs
Total Cholesterol	rs7033776	*CLTA, CREB3, FBXO10, FRMPD1, GBA2, GLIPE2, GNE, GRHPR, HINT2, MELK, MSMP, NRP2, PAX5, POLR1E, RECK, RGP1, SPAG8, TLN1, TMEM8B, TOMM5, ZBTB5, ZCCHC7*
BMI	rs77785972	*CHD1, RGMB, RGMB-AS1, RIOK2*
	rs17605739	-
LDL	rs1032596	*C16orf74, C16orf95, COX4I1, GINS2, IRF8, MTHFSD*
Triglycerides	rs7527236	*LYPLA1, RRP15, TGFB2*
LVMI	rs16853098	*STK39*
Age at menarche	rs58758366	-
Eosinophil count	rs2355879	*AKR7A2, ALDH4A1, ARHGEF10L, CAPZB, IFFO2, MRTO4, PQLC2, RCC2, UBR4*
Neutrophil count	rs142030841	*C18orf21, CELF4, ELP2, FHOD3, KIAA1328, MOCOS, RPRD1A, SLC39A6, TPGS2*
FEV_1	rs12714812	*FAM86DP*

Supplementary Table 4. Candidate Genes for Parent of Origin Differential eQTL.

Phenotype	SNP	GWAS	p-value
SBP	rs1536182	Maternal	0.970
		Paternal	0.471
Age of menarche	rs7184983	Maternal	0.600
		Paternal	0.391
LDL	rs4843650	Maternal	0.288
		Paternal	0.938
	rs12024326	Maternal	0.154
		Paternal	0.684
Total cholesterol	rs113588203	Maternal	0.186
		Paternal	0.129
CIMT	rs4077567	Maternal	0.833
		Paternal	0.363
FEV1	rs6791779	Maternal	1.0
		Paternal	0.800
	rs9849387	Maternal	0.0391
		Paternal	0.337
BMI	rs77785972	Opposite effect	7.7e-05
	rs17605739	Opposite effect	0.816
Total Cholesterol	rs7033776	Opposite effect	0.583
Eosinophil count	rs2355879	Opposite effect	0.39
Triglycerides	rs7525463	Opposite effect	0.0396
FEV_1	rs12714812	Opposite effect	0.35
LVMI	rs16853098	Opposite effect	0.335
Neutrophil count	rs142030841	Opposite effect	0.092
Age of menarche	rs12447191	Opposite effect	0.70

Supplementary Table 5. Replication in Sardinia

rsid	bp	chr	Maternally inherited allele frequency	Paternally inherited allele frequency
rs2355879	18732860	1	0.1289	0.1289
rs574232282	41662388	1	0.01465	0.1465
rs7525463	218860879	1	0.1859	0.1208
rs12024326	227146433	1	0.1845	0.1651
rs113588203	228979156	1	0.1058	0.08989
rs16853098	168013281	2	0.126	0.125
rs4077567	216703202	2	0.2726	0.2826
rs12714812	74813002	3	0.4204	0.4532
rs6791779	74996505	3	0.2392	0.2049
rs9849387	77764243	3	0.4232	0.3689
rs77785972	97415767	5	0.02268	0.02268
rs17605739	22962798	6	0.1884	0.1453
rs7033776	36704465	9	0.4521	0.3842
rs1536182	46275415	13	0.1911	0.1855
rs7184983	56554709	16	0.04135	0.07692
rs12447191	62199299	16	0.1544	0.2096
rs1032596	86281537	16	0.3066	0.3515
rs4843650	87683486	16	0.4251	0.4934
rs142030841	34371947	18	0.02372	0.06834

Supplementary Table 6. Parental allele frequency of significant single parent and opposite effect parental associations from Table 1 and Table 2.
